# An EHMT2/NFYA-ALDH2 signaling axis modulates the RAF pathway to regulate paclitaxel resistance in lung cancer

**DOI:** 10.1186/s12943-022-01579-9

**Published:** 2022-04-27

**Authors:** Wenjing Wang, Jianmin Wang, Shuai Liu, Yong Ren, Jingyu Wang, Sen Liu, Wei Cui, Lina Jia, Xing Tang, Jingyu Yang, Chunfu Wu, Lihui Wang

**Affiliations:** 1 Department of Pharmacology, Shenyang Pharmaceutical University, Shenyang, People’s Republic of China; 2 Benxi Institute of Pharmaceutical Research, Shenyang Pharmaceutical University, Shenyang, People’s Republic of China; 3 Department of Pathology, General Hospital of Central Theater Command of People’s Liberation Army, Wuhan, People’s Republic of China; 4 Department of Pharmaceutics, Shenyang Pharmaceutical University, Shenyang, People’s Republic of China

**Keywords:** ALDH2, EHMT2, Non-small cell lung cancer, Paclitaxel resistance, RAS/RAF pathway

## Abstract

**Background:**

Lung cancer is a kind of malignancy with high morbidity and mortality worldwide. Paclitaxel (PTX) is the main treatment for non-small cell lung cancer (NSCLC), and resistance to PTX seriously affects the survival of patients. However, the underlying mechanism and potential reversing strategy need to be further explored.

**Methods:**

We identified ALDH2 as a PTX resistance-related gene using gene microarray analysis. Subsequently, a series of functional analysis in cell lines, patient samples and xenograft models were performed to explore the functional role, clinical significance and the aberrant regulation mechanism of ALDH2 in PTX resistance of NSCLC. Furthermore, the pharmacological agents targeting ALDH2 and epigenetic enzyme were used to investigate the diverse reversing strategy against PTX resistance.

**Results:**

Upregulation of ALDH2 expression is highly associated with resistance to PTX using in vitro and in vivo analyses of NSCLC cells along with clinicopathological analyses of NSCLC patients. ALDH2-overexpressing NSCLC cells exhibited significantly reduced PTX sensitivity and increased biological characteristics of malignancy in vitro and tumor growth and metastasis in vivo. EHMT2 (euchromatic histone lysine methyltransferase 2) inhibition and NFYA (nuclear transcription factor Y subunit alpha) overexpression had a cooperative effect on the regulation of ALDH2. Mechanistically, ALDH2 overexpression activated the RAS/RAF oncogenic pathway. NSCLC/PTX cells re-acquired sensitivity to PTX in vivo and in vitro when ALDH2 was inhibited by pharmacological agents, including the ALDH2 inhibitors Daidzin (DZN)/Disulfiram (DSF) and JIB04, which reverses the effect of EHMT2.

**Conclusion:**

Our findings suggest that ALDH2 status can help predict patient response to PTX therapy and ALDH2 inhibition may be a promising strategy to overcome PTX resistance in the clinic.

**Supplementary Information:**

The online version contains supplementary material available at 10.1186/s12943-022-01579-9.

## Introduction

Lung cancer is a kind of malignancy with high morbidity and mortality worldwide, it is responsible for more than 1.7 million deaths each year [1]. About 80–85% of lung cancers are classified pathologically as non-small cell lung cancer (NSCLC) [2]. Paclitaxel (PTX) interferes with microtubule dynamics, and is a first-line chemotherapeutic drug for the treatment of advanced NSCLC [3]. However, with prolongation of treatment time, the patient can easily develop resistance to PTX, which reduces its efficacy. At present, the common PTX resistance mechanisms are as follows: 1) overexpression of certain transmembrane efflux transporters, such as ABCB1 and ABCC10 [4, 5]; 2) dynamic changes in the stability of microtubules, such as β-tubulin binding changes [6–8]; 3) changes in the function of apoptosis-related proteins, such as Bcl-2 [9–12], etc. However, therapeutic approaches based on these mechanisms have not achieved the promised efficacy. Therefore, there is an urgent need to explore the molecular mechanism of PTX resistance and discover new drug resistance targets and therapeutic drugs.

Epigenetic modification is a kind of biological process in which the DNA sequence does not change but the phenotype changes [13]. Epigenetic changes are reversible and mainly include DNA methylation modification and histone modification (methylation, acetylation) [14]. It has been reported that the characteristics of epigenetic regulation are similar to drug resistance. For example, both processes are independent of genotype changes [15], especially in chemotherapy resistance. Furthermore, both processes are reversible; it has been shown that both TKI resistance and chemotherapy resistance have the characteristics of self-reversal [16, 17]. Therefore, there might be a connection between drug resistance and epigenetic regulation. Indeed, several studies from our lab and another group have confirmed the relationship between epigenetic regulation and drug resistance [18, 19]. However, it is still not known whether epigenetic regulations play a role in PTX resistance in lung cancer.

The acetaldehyde dehydrogenase (ALDH) gene family encodes 19 enzymes, which possess important physiological and toxicological functions [20]. Among the 19 human ALDH subtypes, ALDH2 is located in the mitochondria, and plays a key role in the metabolism of ethanol-derived acetaldehyde [21, 22]. Furthermore, ALDH2 is widely used as a marker of cancer stem cells (CSCs) [23]. It is reported that ALDH2 is involved in tumor drug resistance. For example, overexpression of ALDH2 resulted in higher cell proliferation rate, higher clone formation rate, and resistance to 4-hydroperoxycyclophosphamide and doxorubicin in leukemia and lung cancer cell lines [24]. In addition, inhibition of ALDH2 with isoflavone glycosides (Daidzin, DZN) and CVT-10216 significantly increased chemotherapy sensitivity in acute myelocytic leukemia cells [25]. However, the relationship between ALDH2 and PTX resistance needs to be further clarified.

In this study, we analyzed the difference in gene expression between PTX-resistant NSCLC cells (NSCLC/PTX cells) and PTX-sensitive NSCLC cells through microarray analysis. We found that ALDH2 expression was upregulated in NSCLC/PTX cells. Next, we dissected the role of ALDH2 in lung cancer resistance to PTX. We revealed the underlying mechanism of epigenetic regulation and we investigated the effects of drug interventions at the molecular, cellular and animal levels. Through this work, we have uncovered a new mechanism of PTX resistance and laid a pharmacological foundation for the discovery of new strategies to reverse drug resistance in lung cancer.

## Materials and methods

### Cell culture and treatment

Human non-small cell lung cancer cell lines NCI-H1299 and NCI-H460 were obtained from ATCC (Gaithersburg, MD, USA). Cells were cultured in RPMI 1640 medium supplemented with 10% FBS (Gibco, Waltham, MA, USA) and 1% penicillin streptomycin (Gibco, Waltham, MA, USA) at 37 °C in 5% CO_2_ incubator. For the generation of the PTX-resistant cell lines, NCI-H1299/PTX and NCI-H460/PTX were cultured in media containing increasing doses of PTX (Med Chem Express, Monmouth Junction, NJ, USA) (from 40 ng/ml to 200 ng/ml) for more than 8 weeks to achieve complete resistance to PTX.

### Microarray and gene expression analysis

Total RNA of NSCLC cells was extracted by TRIzol Reagent (Invitrogen, Waltham, MA, USA) as described in the manufacturer’s instructions. The RNA was hybridized to an array (Clariom D assay) and the hybridized array was scanned with an Affymetrix Genechip Scanner. The raw microarray data of CEL files were normalized by Robust Multichip Average (RMA) assay.

### Clonogenic assay

Cells (3 × 10^2^/well) were plated in a 6-well plate and the culture medium was replaced every 2 days. After culturing for 7–10 days, cells were fixed with 4% paraformaldehyde for 15 min and stained with 0.1% crystal violet for 30 min, and the colonies were imaged and counted.

$$\mathrm{Clone formation rate }=\mathrm{ Number of clones }/ 300 * 100\mathrm{\%}$$

### Tumor sphere formation assay

Cells were plated at 3 × 10^3^ cells/ml in ultra-low attachment 6-well plates (Corning, NY, USA) in serum-free DMEM/F12 supplemented with B27 (1:50), 20 ng/ml EGF and 20 ng/ml βFGF. Every 3 days, 500 μl of fresh medium was added. After 10 days, tumor spheres were photographed under a microscope with the bright-field.

$$\mathrm{Tumor sphere formation rate }=\mathrm{ Number of tumor spheres }/ 3000 * 100\mathrm{\%}$$

### Transwell migration assays

Pre-starved cells were plated in the upper Transwell chamber and incubated for 24 h at 37 °C in 5% CO_2_. Non-migrated cells were scraped from the upper surface of the membrane with a cotton swab, and migrated cells remaining on the bottom surface of the membrane were stained with calcein-AM for 15 min and photographed using an inverted fluorescence microscope.

$$\mathrm{Migration rate }=\mathrm{ Number of migrated PTX}-\mathrm{resistant cells }/\mathrm{ Number of migrated parental cells }* 100\mathrm{\%}$$

### Western blot analysis

Cells were lysed with RIPA lysis buffer (Cell Signaling Technology, Danvers, MA, USA) containing phosphatase inhibitor and protease inhibitor cocktail (Med Chem Express, Monmouth Junction, NJ, USA). Total protein was separated on sodium dodecyl sulphate–polyacrylamide gel electrophoresis (SDS-PAGE) gels and then transferred onto polyvinylidene fluoride (PVDF) membranes. The membranes were blocked with 5% nonfat dried milk for 1 h at room temperature and incubated with the indicated primary antibody overnight at 4 °C, followed by incubation with secondary antibodies for 1 h at room temperature. The protein bands were visualized with ECL detection reagents (Thermo Fisher Scientific, Waltham, MA, USA). All antibody information is shown in Supplementary Table 1.

### Flow cytometric analysis of ALDH activity

The ALDH enzyme activity was measured by flow cytometry using an Aldefluor kit (StemCell Tech., Durham, NC, USA) following the manufacturer’s instructions. Diethylamino-benzyaldehyde (DEAB), a specific ALDH inhibitor, was used as a control. Cells (1 × 10^6^/ml) were collected and stained for 30 min at 37℃. Cell with ALDH activity had greater fluorescence than cells in which the enzyme activity was inhibited by DEAB.

### Generation of stable cell lines using lentivirus

To create stable NSCLC/PTX cell lines, shRNA-expressing vector was introduced by lentiviral infection. For preparation of viruses, HEK293T cells were transfected using lipofectamine 3000 (Thermo Fisher Scientific, Waltham, MA, USA) with a 4:3:2 ratios of shRNA construct: psPAX2: pMD2.G in opti-MEM solution. Briefly, 10 μg of the shRNA plasmid, 7.5 μg of psPAX2, and 5 μg of pMD2.G were transfected into 293 T cells plated in a 100 mm dish. Viral supernatant was collected 48 h and 72 h post-transfection, centrifuged at 3,000 rpm for 30 min to remove 293 T cells, and filtered (0.45 μm). For transduction, viral supernatants were used to infect NSCLC cells with 8 μg/ml polybrene; 48 h after infection, cells were selected using puromycin and tested by real-time PCR or western blot.

### MTT assay

Cells (4 × 10^3^/well) were seeded into a 96-well plate and cultured at 37 °C in 5% CO_2_ incubator. The concentrations of PTX ranged from 0.01 μM to 100 μM with a minimum of three technical replicates. At the final time point, 10 μl MTT solution (5 mg/ml, Sigma, Burlington, MA, USA) was added to each well and incubated for an additional 4 h at 37 °C in 5% CO_2_ incubator. Then, 100 μl DMSO was added to each well and mixed thoroughly. The optical density values were measured at 492 nm using a microplate reader (Molecular Devices, San Jose, CA, USA).

### RNAi transfection

Cells were transfected with control siRNA or specific siRNA when confluence reached 70–90%, and transfection was performed using lipofectamine 3000 (Thermo Fisher Scientific, Waltham, MA, USA) at a final concentration of 20 nM according to the manufacturer's instructions. After transfection for 48 h, cells were subjected to MTT assay, and the expression levels of specific proteins were measured by western blot. The siRNAs to ALDH1A1 (4,390,824, ID: s1236), ALDH3A1 (4,390,824, ID: s1242) and ALDH2 (4,390,824, ID: s1239) were purchased from Thermo Fisher Scientific (Waltham, MA, USA). The other ALDH2 (217–1) siRNA was purchased from BIONEER (Daejeon, Korea).

### In vivo metastatic model and bioluminescent imaging

For the tail vein injection model, 1 × 10^6^ luciferase-expressing NSCLC cells with the indicated modification were injected into tail veins of mice. Each mouse was intraperitoneally injected with 200 mg/kg D-Luciferin, and development of metastases was monitored by bioluminescence imaging (BLI).

### RNA extraction and quantitative real-time PCR analysis

Total RNA was extracted using Trizol and transformed to cDNA using an All-in-One First-Strand cDNA Synthesis Kit (Transgene, Beijing, China) following the manufacturer’s instructions. Quantitative real-time PCR analysis was performed by mixing 10 μl SYBR, 0.4 μM forward primer, 0.4 μM reverse primer, 2 μl cDNA, and 7.2 μl distilled water per sample with Top Green qPCR SuperMix (Transgene, Beijing, China) in accordance with the manufacturer’s protocols. β-Actin was useed as the endogenous controls. The relative levels of PCR products were calculated according to the following equation: Relative quantity = 2^−ΔΔCt^. The primer sequences utilized in this research are displayed in Supplementary Table 2.

### Luciferase reporter assay

pGL3-Basic (Fluc) and phRL-TK (Rluc) plasmids were purchased from Promega (Madison, WI, USA). ALDH2 promotor fragments were inserted into the pGL3-Basic vector to generate PGL3-ALDH2-P1, PGL3-ALDH2-P2 and PGL3-ALDH2-P3. Cells were transfected with these plasmids, together with the equivalent Renilla luciferase, followed by gene overexpression or drug treatment. Cells were harvested 24 h after transfection, and luciferase activity was measured by Dual-Luciferase Reporter Assay kits (Promega, Madison, WI, USA) in accordance with the manufacturer’s protocols. Luciferase activities were normalized to Renilla luciferase activity. Binding sites for the transcription factors HNF4A and NFYA were mutated based on a consensus nucleotide sequences in the ALDH2 P1 promoter. The resulting constructs were pGL3-ALDH2-HNF4A-M1/M2/M3 and pGL3-ALDH2-NFYA-M1/M2.

### ChIP-sequencing assay

Genome-wide profiling of the histone marks H3K9me2 and Ac-H3 was performed using the SimpleChIP Plus Sonication Chromatin Immunoprecipitation Kit (Cell Signaling Technology, Danvers, MA, USA) as described in the manufacturer’s instruction. In brief, cells were cross linked by polyoxymethylene and chromatin was sonicated into several small fractions. Then, a specific antibody was used to bind histones carrying H3K9me2 and Ac-H3, and the bound DNA was purified from the proteins. The bound DNA was analyzed by next- generation sequencing technology.

### Chromatin immunoprecipitation (ChIP)

ChIP assays were performed using the Chromatin Immunoprecipitation Kit (Cell Signaling Technology, Danvers, MA, USA) according to the manufacturer’s protocol. Briefly, NCI-H460 cells (1 × 10^7^) were fixed with cross-linking solution and collected. Samples were sonicated and DNA was sheared to an average length of approximately 250–450 bp. DNA–protein complexes were immunoprecipitated using 5 μg of anti-NFYA, anti-H3K9me2, and anti-AcH3 antibodies or with polyclonal IgG control at 4 °C overnight. Immunoprecipitated DNA was analyzed by quantitative PCR.

### In vivo tumour xenograft animal model

4- to 6-week-old BALB/c nude mice were maintained in a specific-pathogen-free (SPF) facility. NCI-H460/PTX cells or luciferase-expressing NSCLC cells with indicated modification (2 × 10^6^ cells in 0.2 ml phosphate-buffered saline) were subcutaneously injected into the right flank of BALB/c nude mice. Tumor volume was measured using calipers every 2 days and was calculated by the following formula: (long diameter) × (short diameter)^2^ /2. For bioluminescent imaging model, each mouse was intraperitoneally injected with 200 mg/kg D-Luciferin and tumor was monitored by bioluminescence imaging (BLI). After the treatments, animals were anesthetized and tumors were excised. Tissues were either fixed in 4% formalin or stored at − 80 °C until further analysis.

$$\mathrm{Viscera index }=\mathrm{ Weight of organ }/\mathrm{ Weight of body }*100\mathrm{\%}$$

### Immunohistochemical (IHC) assay and TUNEL assay

Clinical tissue samples and tumor samples acquired from the mice bearing H460/PTX cells were embedded in paraffin and antigen retrieval was performed. Following the blockade of endogenous peroxidase activity, samples were incubated with the primary antibodies of interest and the appropriate secondary antibodies and reacted with DAB detection reagents. The immunoreactive staining of proteins in tumor tissue was scored by applying a semiquantitative immunoreactive scoring (IRS) system as previously reported [19]. The median value of the immunoreactive score was chosen as the cut-off criterion to dichotomize into high- and low-expression subgroups.

TUNEL assays were performed for quantification of apoptosis. TUNEL staining was performed using the TUNEL Assay Kit—HRP-DAB (Abcam, Cambridge, UK), according to the manufacturer’s recommended protocol.

### Statistical analysis

Statistical analyses were performed using SPSS v.26.0 (SPSS). Data in all graphs are represented as mean ± SD of biological triplicates. Statistical significance was determined by Student’s t test, one-way ANOVA or two-way ANOVA. For all statistical tests, the 0.05 level of confidence was accepted for statistical significance.

## Results

### Identification of ALDH2 as a paclitaxel resistance-related gene in NSCLC cells

**Fig. 1:**
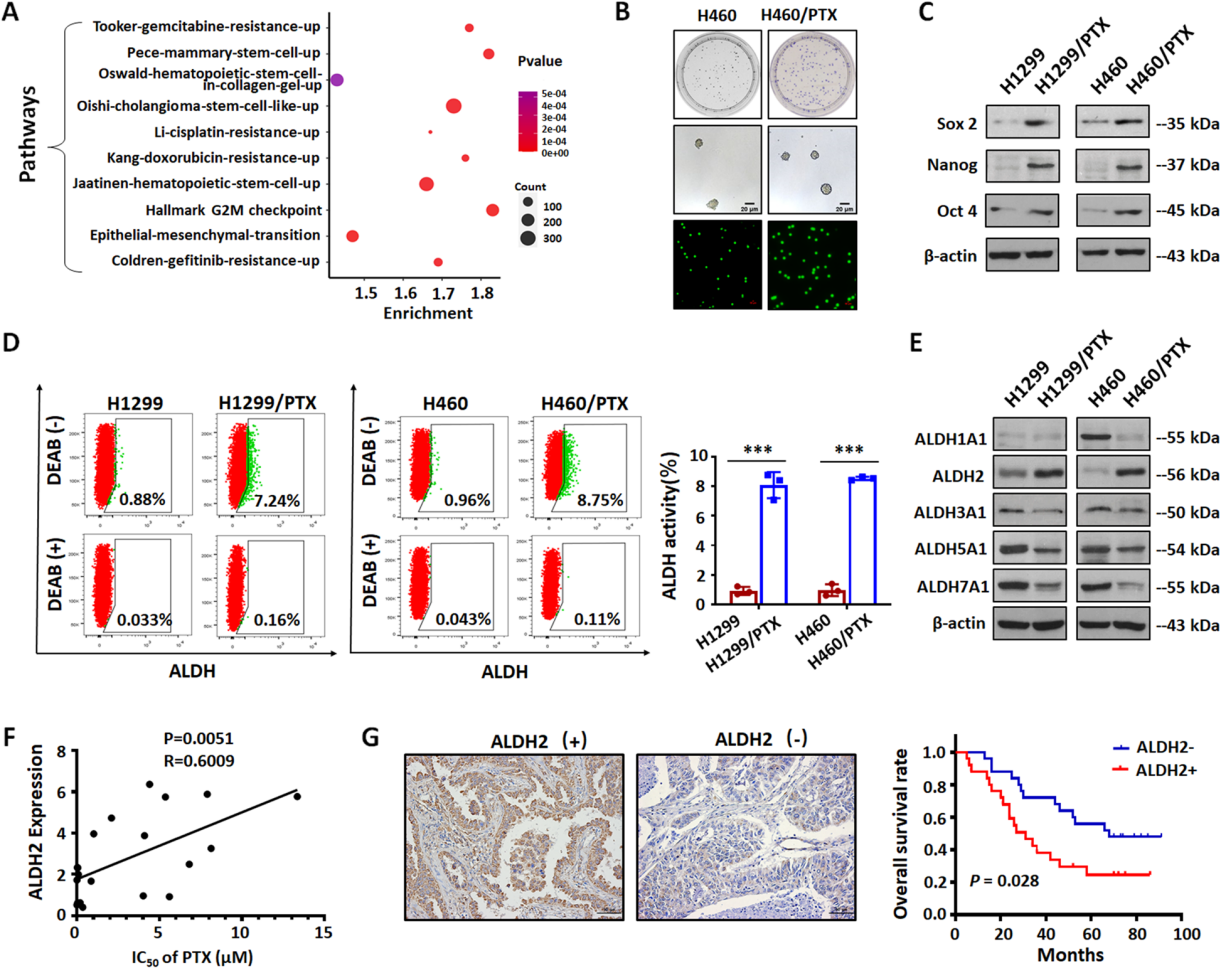
Identification of ALDH2 as a paclitaxel resistance-related gene in NSCLC. **A** Bubble diagram of KEGG pathway analysis based on the differentially expressed genes in NCI-H460/PTX cells compared with NCI-H460 cells (three biological replicates for each condition). The diagram shows enriched pathways. **B** Colony formation assay, tumor sphere formation assay and Transwell migration assay in NCI-H460 and NCI-H460/PTX cells. The photographs were taken at magnifications of × 200. Scale bar = 50 μm. **C** The protein expression levels of the stem cell transcription factors Sox2, Nanog, and Oct4 in NSCLC and NSCLC/PTX cells. **D** ALDH activity was determined in NSCLC and NSCLC/PTX cells. Cells were labeled with Aldefluor with or without the ALDH inhibitor DEAB and analyzed by flow cytometry. **E** The protein expression levels of ALDH subtypes in NSCLC and NSCLC/PTX cells. **F** The relationship between ALDH2 expression level and PTX resistance in lung cancer cells. **G** Correlation of the expression of ALDH2 protein with overall survival. ^***^p < 0.001, as compared to parental cells

We first measured the resistance index (RI) value of the NSCLC/PTX cells to confirm PTX resistance. Results showed that the PTX resistance index of NSCLC/PTX cells is greater than 15 (Fig. S1A). Thus, the cells are highly drug-resistant and can be used in subsequent experiments. In order to confirm the mechanism of PTX resistance in NSCLC, we performed gene microarray analysis in NCI-H460 and NCI-H460/PTX cells to investigate the differentially expressed genes. Pathway analysis showed that the pathways related to stemness and drug resistance were significantly enriched (*P* < 0.05), suggesting that stemness changes may be involved in PTX resistance in lung cancer (Fig. 1A). Next, we further analyzed the biological characteristics of NSCLC cells and NSCLC/PTX cells. Results from clone formation experiments, sphere formation experiments, and Transwell migration experiments indicated that the self-renewal and migration capabilities of NSCLC/PTX cells were significantly increased compared with NSCLC cells (Fig. 1B; Figs. S1B and C). The above results suggested that the biological stem cell-like characteristics of NSCLC/PTX cells were enhanced. Next, we analyzed the protein expression of stem cell transcription factors. The results indicated that the expression of Sox2, Nanog and Oct4 in NSCLC/PTX cells was higher than NSCLC cells (Fig. 1C).

ALDH is widely used as a marker of cancer stem cells (CSCs) [26]. We found that the ALDH activity of NSCLC/PTX cells was significantly higher than that of NSCLC cells (Fig. 1D). Taken together, the above results suggested that the stemness characteristics of NSCLC/PTX cells were increased compared with NSCLC cells. Further analysis indicated that among ALDH subtypes, the protein expression of ALDH2 was higher in NSCLC/PTX cells than NSCLC (Fig. 1E). In order to further explore whether ALDH2 is involved in the PTX resistance process in lung cancer, we used the Cancer Cell Line Encyclopedia (CCLE) and Genomics of Drug Sensitivity in Cancer (GDSC) database to analyze the relationship between the two. The results showed that the expression level of ALDH2 is significantly related to the sensitivity to PTX. The higher the expression level of ALDH2, the less sensitive the cells are to PTX (Fig. 1F; Fig. S1D). Importantly, in order to confirm the relationship between ALDH2 and PTX sensitivity in NSCLC patients, we determined ALDH2 expression levels in tissue specimens from 46 NSCLC patients with PTX-based treatment. The results indicated that high expression levels of ALDH2 protein were significantly correlated with poor overall survival compared to the low ALDH2 groups (Fig. 1G). Taken together, the above results suggested that ALDH2 plays an important role in the accumulation of stemness characteristics and the process of PTX resistance in NSCLC cells.

### The efficacy of PTX is altered when ALDH2 is regulated by pharmacological inhibitors or genetic intervention in NSCLC cells

**Fig. 2:**
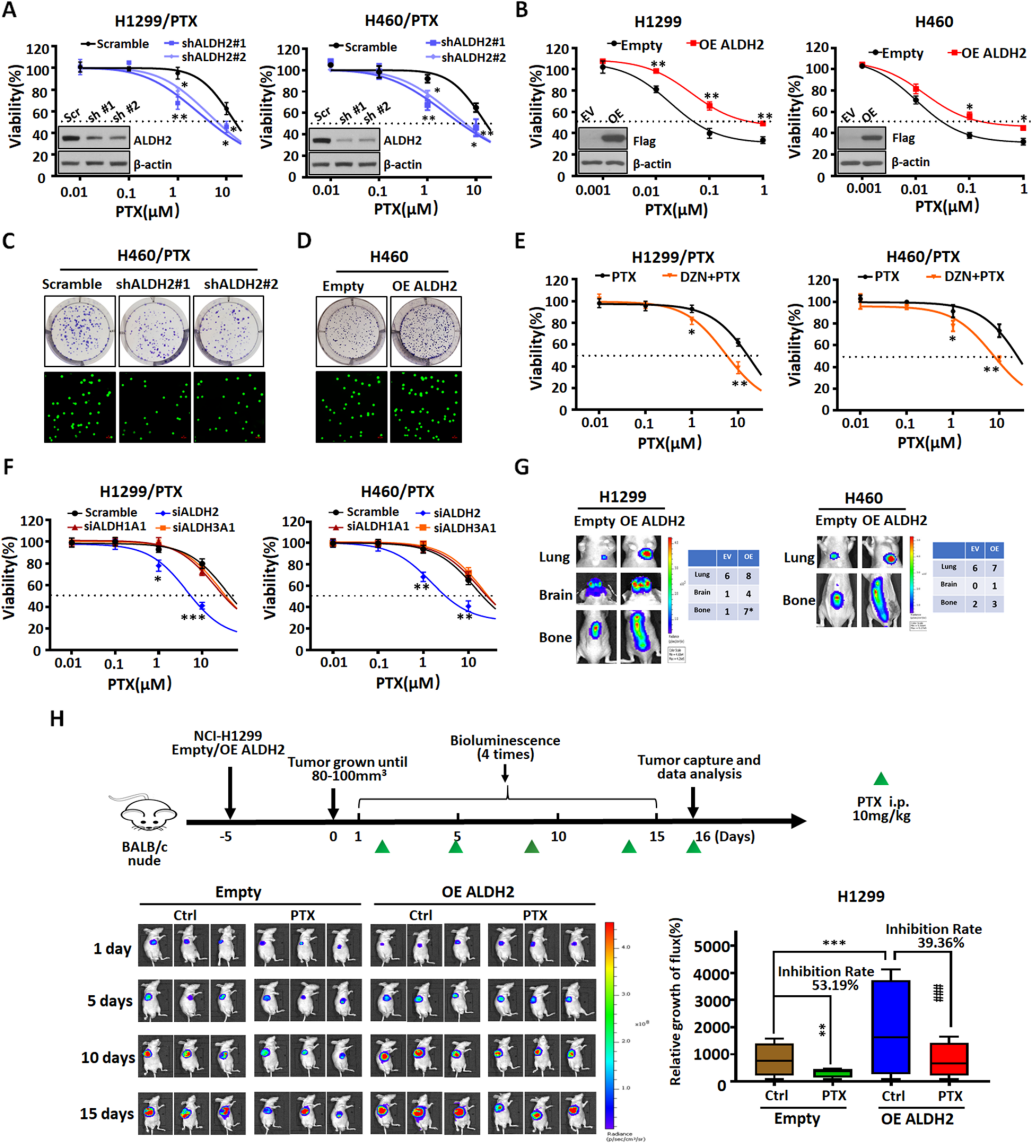
The efficacy of PTX is altered when ALDH2 is regulated by drug or genetic intervention in NSCLC cells. **A** MTT assay results showing the efficacy of PTX in NSCLC/PTX cells transfected with ALDH2 shRNA or scramble for 72 h. ^*^p < 0.05, ^**^p < 0.01, as compared to the scramble group. **B** MTT assay results showing the efficacy of PTX in NSCLC cells transfected with ALDH2 overexpression (OE) or empty plasmid for 72 h. ^*^p < 0.05, ^**^p < 0.01, as compared to the empty group. **C** Colony formation assay and Transwell migration assay in NCI-H460/PTX cells transfected with ALDH2 shRNA or scramble. The photographs were taken at magnifications of × 200. Scale bar = 50 μm. **D** Colony formation assay and Transwell migration assay in NCI-H460 cells transfected with ALDH2 overexpression (OE) or empty plasmid. The photographs were taken at magnifications of × 200. Scale bar = 50 μm. **E** MTT assay results showing the efficacy of PTX in NSCLC/PTX cells treated with DZN (10 μM) or vehicle for 72 h. ^*^p < 0.05, ^**^p < 0.01, as compared to the PTX group. **F** MTT assay results showing the efficacy of PTX in NSCLC/PTX cells transfected with siRNAs against specific ALDH subtypes or control siRNA for 72 h. ^*^p < 0.05, ^**^p < 0.01, ^***^p < 0.001, as compared to the scramble group. **G** The number of metastases from NSCLC cells transfected with ALDH2 overexpression (OE) or empty plasmid in lung, brain and bone. **H** Top**:** Schematic view of the establishment and treatment of NCI-H1299 xenograft mice. NCI-H1299 cells were injected into the right flank of BALB/c nude mice. PTX was administered as indicated by colored triangles (i.p., intraperitoneal). Bottom: Bioluminescence images showing tumor growth in NCI-H1299 xenograft mice treated with PTX or vehicle. ^**^p < 0.01, ^***^p < 0.001, as compared to the empty group, ^###^*p* < 0.001, as compared to the ALDH2 overexpression group

In order to further clarify the relationship between ALDH2 and PTX resistance, we determined the efficacy of PTX treatment in NSCLC cells and NSCLC/PTX cells after ALDH2 manipulation. Indeed, the efficacy of PTX was significantly increased in the NSCLC/PTX cells after the knockdown of ALDH2, whereas the efficacy of PTX was significantly decreased in NSCLC cells overexpressing ALDH2 (Fig. 2A-B). To clarify the role of ALDH2, we performed clonogenic assays and migration assays in NSCLC/PTX cells and NSCLC cells with stable knockdown or overexpression of ALDH2. Following ALDH2 knockdown, the ability of NSCLC/PTX cells to undergo migration and self-renewal was significantly decreased (Fig. 2C; Fig. S2A-B). The migration and self-renewal ability of NSCLC cells was significantly increased after overexpressing ALDH2 (Fig. 2D; Fig. S2C-D). Daidzin (DZN), a specific inhibitor of ALDH2 (Fig. S2E), significantly inhibited ALDH activity at non-cytotoxic concentrations in NSCLC/PTX cells (Fig. S2F). In addition, DZN in combination with PTX at a non-cytotoxic concentration significantly increased the sensitivity of NSCLC/PTX cells to PTX (Fig. 2E). Next, in order to further verify the specificity of relationship between ALDH2 and PTX resistance, we transiently silenced ALDH2 and other CSCs-related ALDH subtypes (ALDH1A1 and ALDH3A1) and determined the sensitivity of NSCLC/PTX cells to PTX. Results indicated that transient silencing of other ALDH subtypes did not significantly affect the sensitivity to PTX. However, when ALDH2 was transiently silenced, the sensitivity to PTX was significantly enhanced in NSCLC/PTX cells (Fig. 2F; Fig. S2G). Taken together, the above results revealed that ALDH2 is an important ALDH subtype that is associated with PTX resistance.

Next, we verified the relationship between ALDH2 and PTX resistance in vivo. First, through lentivirus transfection, we created NCI-H1299 and NCI-H460 cells expressing luciferase alone (empty vector group) or luciferase + ALDH2 (ALDH2 overexpression group). Balb/c-nu mice were then injected with the cells via the tail vein. The results indicated that mice injected with ALDH2-overexpressing cells have a higher lung, brain, and bone metastasis rate compared with the empty vector groups (Fig. 2G). In addition, in vivo data indicated that NCI-H1299 xenograft tumors where ALDH2 was overexpressed were more resistant to PTX (inhibition rate 39.36%) than control NCI-H1299 xenograft tumors without ALDH2 overexpression (inhibition rate 53.19%) (Fig. 2H). Similarly, conclusions were drawn in the NCI-H460 xenograft tumors model (Fig. S2H). Taken together, the above results indicated that ALDH2 is related to PTX resistance in vivo.

### The transcription factor NFYA is involved in the transcriptional activation of ALDH2

The gene microarray results indicated that the expression of ALDH subtypes changed in NSCLC/PTX cells. Notably, the expression of ALDH2 increased (Fig. S3A). In order to clarify the mechanism of ALDH2 dysregulation, we performed RT-PCR analysis on NSCLC cells and their PTX-resistant derivatives (Fig. S3B). The results confirmed that the transcription level of ALDH2 changed. In addition, we investigated the translation and proteasome degradation pathways. The protein expression of ALDH2 did not change in NSCLC and NSCLC/PTX cells treated with Cycloheximide (CHX) or MG132 (Fig. S3C). The above results suggested that the altered ALDH2 transcription level is the main mechanism leading to ALDH2 dysregulation.

**Fig. 3:**
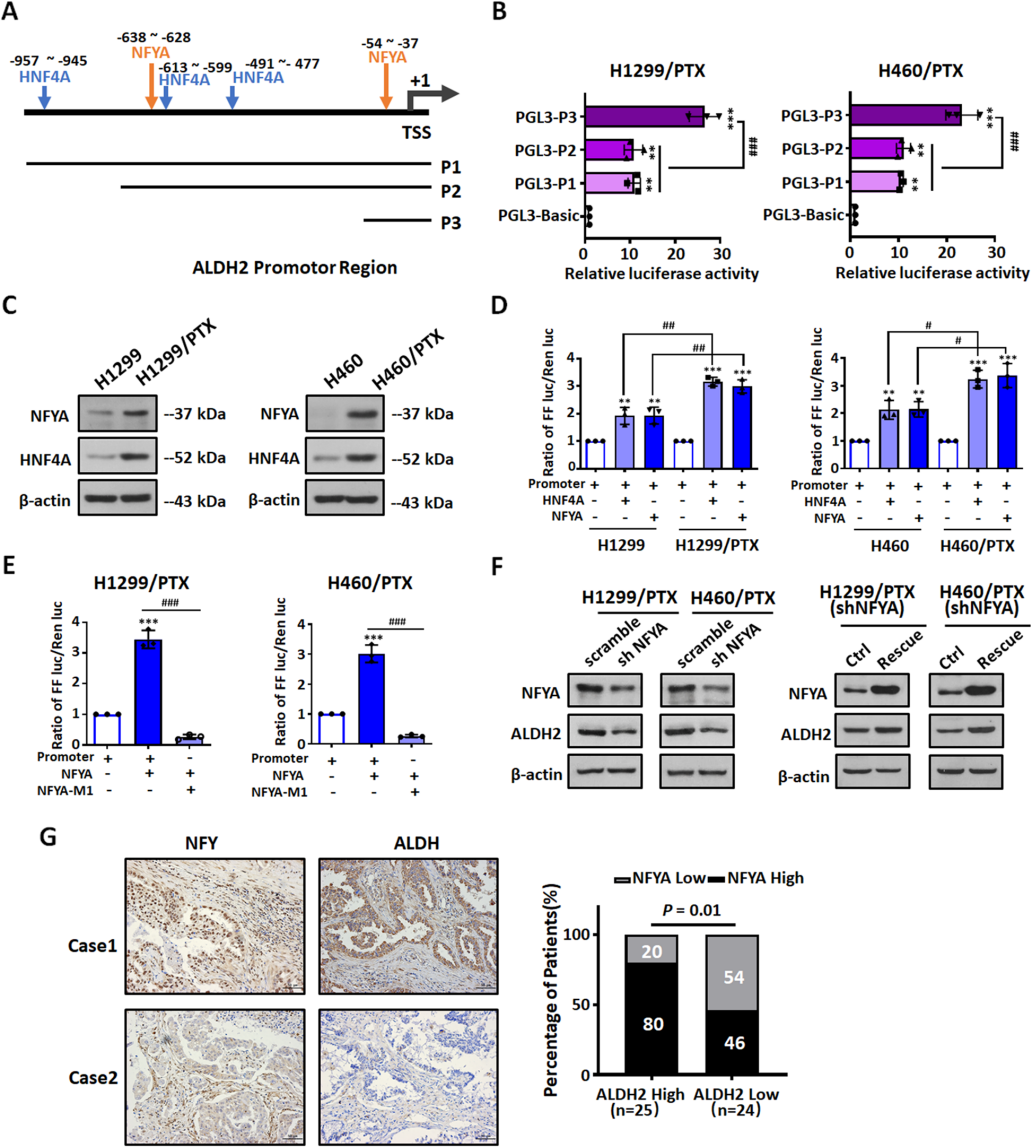
The transcription factor NFYA is involved in the transcriptional activation of ALDH2. **A** Schematic diagram of the ALDH2 promotor region (P1) showing the location of predicted binding sites for the transcription factors, HNF4A and NFYA. The promoter deletions (P2-P3) are shown below. **B** Dual luciferase reporter assay for the transcriptional activity of three ALDH2 promoter fragments (P1-P3) in NSCLC/PTX cells. ^**^p < 0.01, ^***^p < 0.001, as compared to the PGL3-Basic group; ^###^*p* < 0.001, as compared to the PGL3-P3 group. **C** The protein expression levels of the NFYA and HNF4A in NSCLC cells and NSCLC/PTX cells. **D** Luciferase activity elicited by the ALDH2 P1 promotor in NSCLC cells and in NSCLC/PTX cells after overexpression of HNF4A and NFYA. ^**^p < 0.01, ^***^p < 0.001, as compared to the promotor group. ^#^*p* < 0.05, ^##^*p* < 0.01, as compared to the parental cells group. **E** Luciferase activity elicited by the NFYA-M1 promoter (the ALDH2 P1 promotor containing the mutated NFYA M1 binding sites, Fig. S4D) in NSCLC/PTX cells after overexpression of NFYA. ^***^p < 0.001, as compared to the P1 promotor group. ^###^*p* < 0.001, as compared to the NFYA overexpression group. **F** The protein expression levels of NFYA and ALDH2 in NSCLC/PTX cells transfected with NFYA shRNA or scramble. **G** Statistical analysis of the expression patterns of NFYA and ALDH2 in tissue specimens from 49 NSCLC patients treated with PTX

Next, we explored the roles of transcription factors and epigenetic modifications upregulating ALDH2 in NSCLC/PTX cells. It was previously reported that transcription factors NFYA [27] and HNF4A [28, 29] bound to the ALDH2 promoter region and activated ALDH2 transcription. We analyzed the promoter region of ALDH2 and predicted the transcription factor binding sites according to PROMO, JASPAR and other bioinformatics databases. Results indicated that there were several binding sites for the transcription factors NFYA and HNF4A (Fig. 3A). We constructed a series of reporter plasmids containing the full-length ALDH2 gene promoter (pGL3-ALDH2-P1) or two deletions (pGL3-ALDH2-P2, P3; Fig. 3A; Fig. S3D). Next, we determined the reporter gene activity in NSCLC and NSCLC/PTX using a dual luciferase reporter assay. Similar patterns of transcriptional activity were seen in both NSCLC cells and NSCLC/PTX cells (Fig. 3B; Fig. S4A). The P3 region, which includes an NFYA binding site, had the highest activity in all four cell lines tested. This suggests that NFYA may act as a transcriptional activator of the ALDH2 promoter. In addition, our data indicated that NSCLC/PTX cells had a higher reporter activity than the parental cells, providing further evidence that ALDH2 plays a role in PTX resistance (Fig. S4A). Next, we determined the protein expression levels of the transcription factors NFYA and HNF4A in NSCLC and NSCLC/PTX cells. The protein levels of NFYA and HNF4A in NSCLC/PTX cells were higher than those in NSCLC cells (Fig. 3C). Further, we investigated the regulatory ability of the transcription factors NFYA and HNF4A on the ALDH2 promoter using a dual luciferase report assay. Results indicated that ALDH2 promoter activity was significantly enhanced in NSCLC and NSCLC/PTX cells transfected with a plasmid overexpressing NFYA or HNF4A (Fig. 3D; Fig. S4B-C). In addition, our data indicated that NSCLC/PTX cells had a higher reporter activity than NSCLC cells when both NFYA and HNF4A were overexpressed. This provides further evidence for the crucial role of both transcription factors in ALDH2 transcriptional regulation.

In order to further confirm the role of NFYA and HNF4A in the regulation of ALDH2, we mutated the NFYA and HNF4A binding sites within the ALDH2 promoter to create the reporter constructs pGL3-ALDH2-NFYA-M1/M2 and pGL3-ALDH2-HNF4A-M1/M2/M3 (Fig. S4D-E) and then determined the transcriptional activity of these reporters in cells. In contrast to other promoter mutation plasmids, the luciferase activity of the pGL3-ALDH2-NFYA-M1 was significantly decreased in NSCLC/PTX cells, which suggests that transcriptional activation of the ALDH2 promoter is dependent on NFYA (Fig. 3E and Fig. S4F). Next we constructed NSCLC/PTX cells with stable knockdown of NFYA and determined protein level of ALDH2. Results indicated that the protein expression level of ALDH2 was decreased in these cells (Fig. 3F). In addition, the protein expression level of ALDH2 was increased when NFYA expression was rescued (Fig. 3F). To confirm the relationship between NFYA and ALDH2, we determined their expression levels in tissue specimens from 49 PTX-treated NSCLC patients using immunohistochemistry. The results indicated that 80% of patients with high levels of NFYA expression (*n* = 20) were in the high ALDH2 expression group (*n* = 25), whereas 54% of patients with lower NFYA expression (*n* = 13) were in the low ALDH2 expression group (*n* = 24). Thus, NFYA expression was positively correlated with the expression of ALDH2 in PTX-treated NSCLC cases (*P* = 0.01, Fig. 3G). Taken together, the above results confirmed that NFYA is an important transcriptional activator of ALDH2.

### The transcriptional activation of ALDH2 is regulated by the cooperation between NFYA and EHMT2

**Fig. 4:**
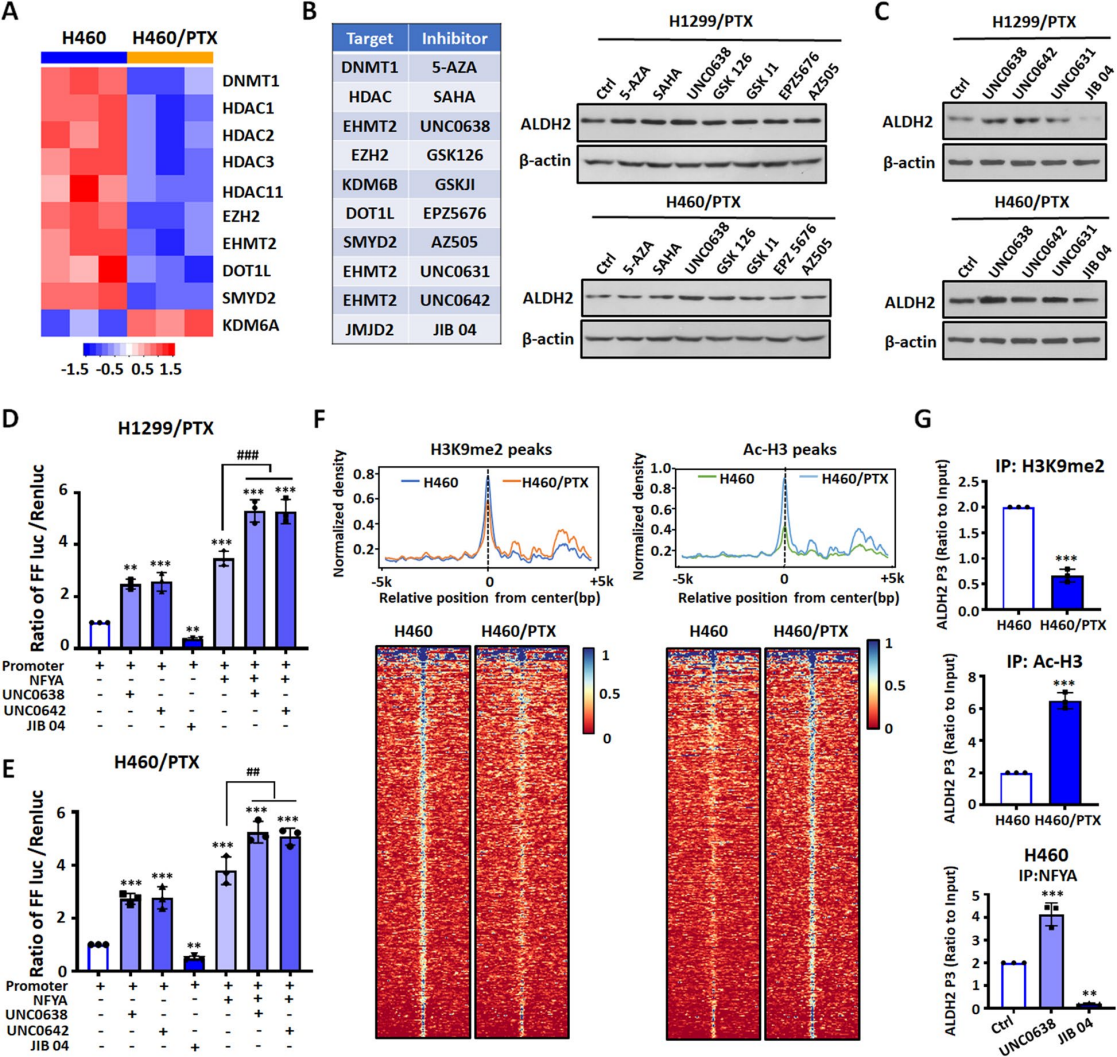
The transcriptional activation of ALDH2 is cooperatively regulated by NFYA and EHMT2. **A** The expression levels of epigenetic enzymes in NCI-H460 and NCI-H460/PTX cells by gene microarray analysis. **B** Left: Epigenetic enzymes and their corresponding inhibitors. Right: The protein expression levels of ALDH2 in NSCLC/PTX cells treated with epigenetic enzyme inhibitors. **C** ALDH2 protein levels in NSCLC/PTX cells treated with EHMT2 inhibitors (UNC0638, UNC0642, UNC0631) and a JMJD inhibitor (JIB04). **D-E** Luciferase activity elicited by the ALDH2 P1 promotor in NSCLC/PTX cells after overexpression of NFYA or treatment with epigenetic enzyme inhibitors. ^**^p < 0.01, ^***^p < 0.001, as compared to the promotor group. ^##^*p* < 0.01, ^###^*p* < 0.001, as compared to the NFYA overexpression group. **F** Left: ChIP-Seq summary plot of H3K9me2-binding intensities across H3K9me2 peaks in NCI-H460 and NCI-H460/PTX cells. Right: ChIP-Seq summary plot of Ac-H3-binding intensities across Ac-H3 peaks in NCI-H460 and NCI-H460/PTX cells. **G** Top: ChIP assay for the H3K9me2 modification involved in ALDH2 in NCI-H460 and NCI-H460/PTX cells. Middle: ChIP assay for the Ac-H3 modification involved in ALDH2 in NCI-H460 and NCI-H460/PTX cells. Bottom: ChIP assay for the NFYA modification involved in ALDH2 in NCI-H460 cells. ^**^p < 0.01, ^***^p < 0.001, as compared to the control group

Epigenetic regulation is closely related to the occurrence and development of tumors. Our gene microarray results revealed that the expression of some epigenetic enzymes was changed in NCI-H460/PTX cells compared with NSCLC cells (Fig. 4A). This indicates that epigenetic mechanisms may be involved in regulation of ALDH2. Therefore, we determined ALDH2 protein levels in NSCLC/PTX cells treated with a series of epigenetic enzyme inhibitors (Fig. 4B). Results indicated that the protein expression of ALDH2 changed to varying degrees. UNC0638, which inhibits EHMT2 (histone lysine methyltransferase 2), obviously upregulated ALDH2 in NSCLC/PTX cells and NSCLC cells (Fig. 4B; Fig. S5A-B). EHMT2 increases the level of H3K9me2, which has an inhibitory effect on transcription. We hypothesized that the expression of ALDH2 would be upregulated when EHMT2 was inhibited. In order to test this hypothesis, we determined ALDH2 protein levels in NSCLC/PTX cells treated with three other EHMT2 inhibitors (UNC0638, UNC0642, UNC0631) and the JMJD inhibitor JIB04. JMJD is a kind of histone demethylase which reduce the level of H3K9me2. As predicted, the EHMT2 inhibitors upregulated the protein expression of ALDH2 and JIB04 had a downregulatory effect (Fig. 4C). These results suggest that EHMT2 and JMJD regulate ALDH2 via an epigenetic mechanism.

In order to verify whether NFYA and EHMT2 have a cooperative effect on the upregulation of ALDH2, we overexpressed NFYA in NSCLC/PTX cells treated with UNC0638, UNCC0642 and JIB04. Results indicated that ALDH2 promoter activity significantly increased when NFYA was overexpressed or when EHMT2 was inhibited; furthermore, NFYA overexpression and EHMT2 inhibition together have a cooperative effect on the upregulation of ALDH2 (Fig. 4D-E). To further understand the epigenetic regulation of ALDH2, we used chromatin immunoprecipitation followed by sequencing (ChIP-seq) to map two histone marks (H3K9me2, Ac-H3) throughout the genome in NCI-H460 and NCI-H460/PTX cells. Results indicated that lower active chromatin signals were observed at H3K9me2 peaks while it is much higher at Ac-H3 peaks in NCI-H460/PTX (Fig. 4F). In addition, we also verified that the accumulation of H3K9me2 was decreased in the ALDH2 promoter region in NCI-H460/PTX compared with NCI-H460, while accumulation of Ac-H3 was increased in the ALDH2 promoter region (Fig. 4G. top and middle). The binding ability of NFYA to the ALDH2 promoter was enhanced in NCI-H460 cells treated with UNC0638, while JIB04 had the opposite effect (Fig. 4G bottom). Overall, these results suggest that NFYA is an important transcriptional activator of ALDH2, and EHMT2 and NFYA have a cooperative effect in the regulation of ALDH2.

### ALDH2 mediated Paclitaxel resistance through RAS/RAF pathway

**Fig. 5:**
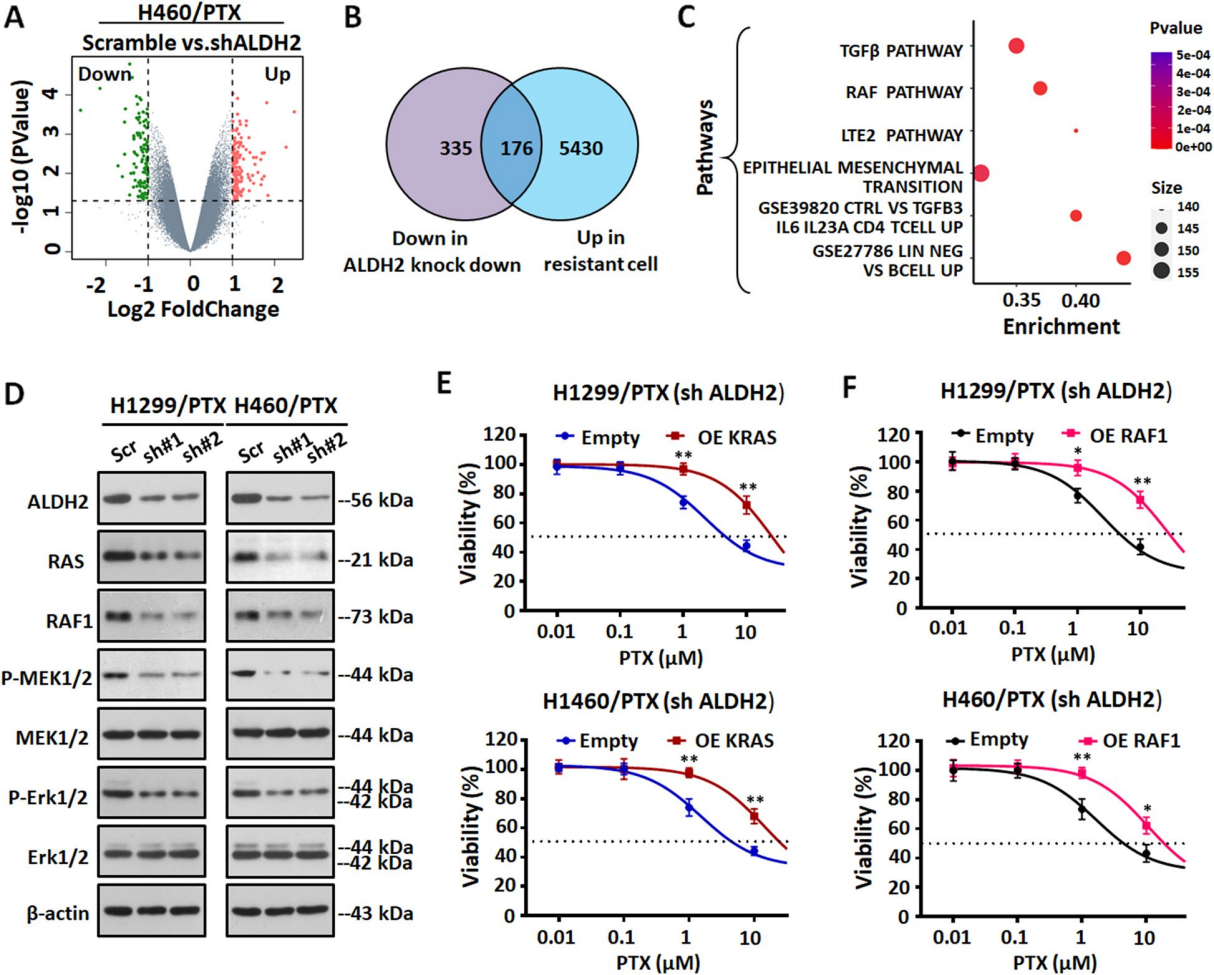
Knockdown of ALDH2 inhibits the RAS/RAF pathway in NSCLC/PTX cells. **A** Volcano plot of gene microarray data illustrating upregulated and downregulated genes of interest (fold change > 2 and *p* < 0.05) in NCI-H460/PTX cells transfected with ALDH2 shRNA compared with scramble (three biological replicates for each condition). **B** Venn diagram showing overlapping genes between upregulated genes in NCI-H460/PTX compared with NCI-H460 and downregulated genes in NCI-H460/PTX transfected with ALDH2 shRNA compared with scramble. **C** Bubble diagram of KEGG pathway analysis based on the differentially expressed genes in NCI-H460/PTX cells transfected with ALDH2 shRNA compared with scramble. Enriched pathways are shown. **D** The expression levels of RAS/RAF pathway-related proteins in NSCLC/PTX cells transfected with ALDH2 shRNA or scramble. **E** MTT assay results showing the efficacy of PTX in ALDH2 knockdown NSCLC/PTX cells transfected with KRAS overexpression (OE) or empty plasmid for 72 h. **F** MTT assay results showing the efficacy of PTX in ALDH2 knockdown NSCLC/PTX cells transfected with RAF1 overexpression (OE) or empty plasmid for 72 h. ^*^p < 0.05, ^**^p < 0.01, as compared to the empty group

In order to elucidate the underlying mechanism of ALDH2-mediated PTX resistance in NSCLC, we performed gene microarray analysis in NCI-H460/PTX cells transfected with ALDH2 or scramble shRNA. The microarray results indicated that many genes were differentially expressed in NCI-H460/PTX cells after stable knockdown of ALDH2; among them, 511 were decreased compared with scramble (Fig. 5A). There were enriched genes between upregulated genes in NCI-H460/PTX compared with NCI-H460 and downregulated genes in NCI-H460/PTX transfected with ALDH2 shRNA compared with scramble (Fig. 5B). Pathway analysis showed that the TGFβ pathway, RAF pathway, EMT pathway and so on were significantly enriched (*P* < 0.05) (Fig. 5C), which suggests that these pathways may be involved in ALDH2-mediated PTX resistance in lung cancer. Next, we examined the levels of proteins in these pathways in ALDH2 knockdown and control (scramble) NSCLC/PTX cells. We first examined TGFβ pathway-related proteins. The results indicated that the levels of P-Smad2 and P-Smad3 did not change consistently in NSCLC/PTX cells (Fig. S6A). Therefore, we think that the TGFβ pathway might not be the main pathway mediating the ALDH2-induced paclitaxel resistance. Next, we investigated the expression of RAS/RAF pathway-related proteins, and we found that their levels were consistently decreased in the ALDH2 knockdown NSCLC/PTX cells compared with the scramble group (Fig. 5D). Therefore, we speculated that the RAS/RAF pathway was involved in ALDH2-mediated paclitaxel resistance. Next, we determined the mRNA levels of downstream targets [30–35] in the RAS/RAF pathways. The mRNA levels of several downstream targets were significantly reduced, which further confirmed the regulation of RAF/RAS pathway by ALDH2 (Fig. S6B). Next, to further clarify whether the RAS/RAF pathway was involved in ALDH2-mediated PTX resistance, we overexpressed KRAS and RAF1, and determined the efficacy of PTX in ALDH2 knockdown NSCLC/PTX cells (Fig. S6C). Results indicated that the efficacy of PTX was decreased when KRAS and RAF1 were overexpressed in ALDH2 knockdown NSCLC/PTX cells (Fig. 5E-F), and the effect was similar to that observed when ALDH2 expression was rescued (Fig. S6D). Taken together, the above results proved that the RAS/RAF pathway is involved in ALDH2-mediated PTX resistance.

### Pharmacological inhibition of ALDH2 sensitizes NSCLC/PTX cells to paclitaxel in vitro and in vivo

**Fig. 6:**
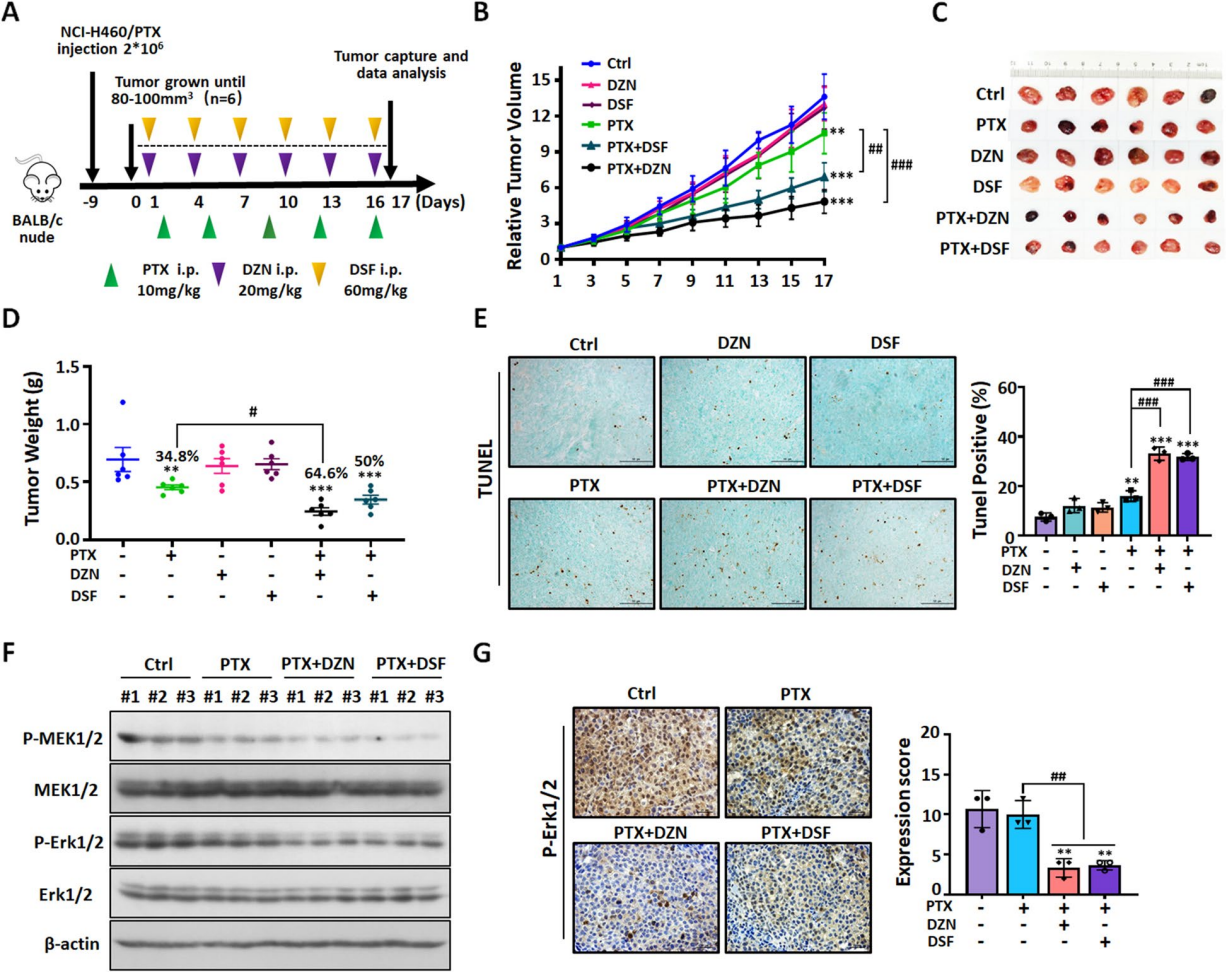
DZN and DSF significantly decrease paclitaxel resistance in NSCLC/PTX cells in vivo. **A** A schematic view of the establishment and treatment of NCI-H460/PTX xenograft mice. NCI-H460/PTX cells were subcutaneously injected into the right flank of BALB/c nude mice. Treatments were administered as indicated by the colored triangles (i.p., intraperitoneal). **B** The inhibitory effect of different treatments on relative tumor volume. **C** Images of tumors from NCI-H460/PTX xenograft mice. **D** The tumor inhibition rate (%) and tumor weight (mg) in the different groups. **E** Images of tumor sections after the TUNEL assay and statistical analysis of TUNEL-positive (apoptotic) cells. The photographs were taken at magnifications of × 200. Scale bar = 50 μm. **F** The expression levels of RAS/RAF pathway-related proteins in NCI-H460/PTX xenograft. **G** Representative immunohistochemistry of P-Erk1/2 in NCI-H460/PTX xenograft and statistical analysis of P-Erk1/2-positive cells. ^**^p < 0.01, ^***^p < 0.001, compared with control group. ^#^*p* < 0.05, ^##^*p* < 0.01, ^###^*p* < 0.001, compared with the PTX group

Our experiments showed that the expression of ALDH2 affects the efficacy of PTX in NSCLC/PTX cells. Therefore, we hypothesized that pharmacological inhibition of ALDH2 may represent a potential approach for the reversal of PTX resistance. DZN is a specific inhibitor of ALDH2 and DSF is a non-specific inhibitor of ALDH2 [26].We investigated the efficacy of PTX combined with DZN or DSF at non-cytotoxic concentrations in NSCLC/PTX cells (Fig. S2E and Fig. S7A). Results indicated that DZN + PTX or DSF + PTX had a synergistic effect on growth inhibition in NSCLC/PTX cells (Fig. S7B-C). In order to validate the above in vitro results, we used the NCI-H460/PTX xenograft model to determine the inhibitory efficacy targeting ALDH2 in vivo (Fig. 6A). Results showed that the tumor volume was significantly smaller in the DZN + PTX group and the DSF + PTX group compared to the PTX alone group (Fig. 6B-C). The tumor weight was significantly lower in DZN + PTX group compared to the PTX alone group (Fig. 6D). These results indicated that targeted inhibition of ALDH2 reverses PTX resistance. Moreover, there was no significant difference between the mice in the PTX group and the combined treatment group in terms of body weight and viscera index (Fig S7D-E). TUNEL staining confirmed that DZN + PTX and DSF + PTX significantly increased the levels of cell apoptosis in tumor tissue compared to PTX alone (Fig. 6E). Furthermore, Western blot results confirmed PTX combined with ALDH2 inhibitors can inhibit Erk1/2 and MEK1/2 phosphorylation compared to PTX alone (Fig. 6F), and immunohistochemical results also confirmed PTX combined with ALDH2 inhibitors can inhibit Erk1/2 phosphorylation compared to PTX alone (Fig. 6G). The above data indicated that pharmacological inhibition of ALDH2 by DZN or DSF can reverse PTX resistance in the NCI-H460/PTX xenograft model by inducing cell apoptosis and inhibiting RAS/RAF pathway.

### Epigenetic downregulation of ALDH2 decreases paclitaxel resistance in NSCLC/PTX cells in vitro and in vivo

**Fig. 7:**
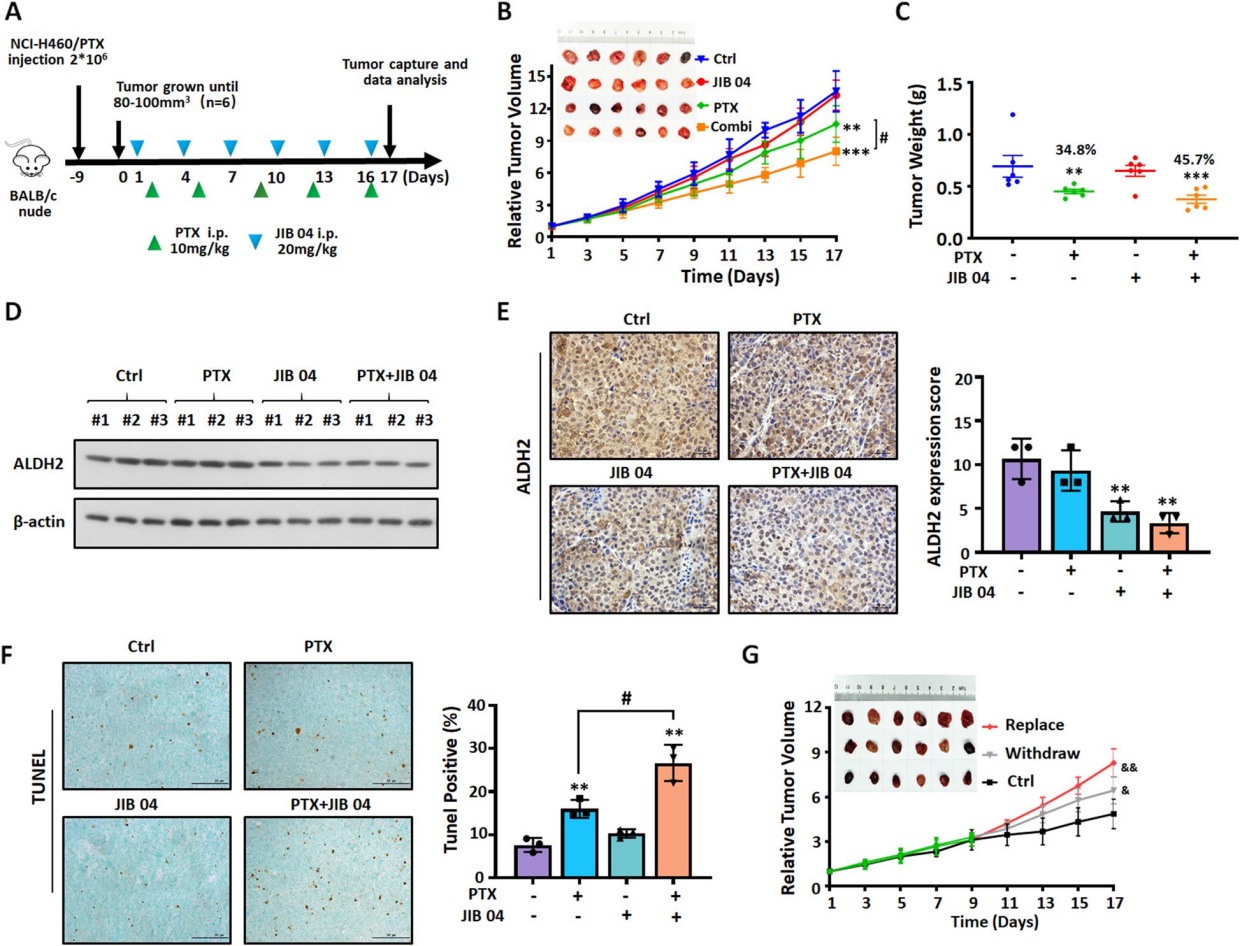
JIB04 significantly decreases paclitaxel resistance in NSCLC/PTX cells in vivo. **A** A schematic view of the establishment and treatment of NCI-H460/PTX xenograft mice. NCI-H460/PTX cells were subcutaneously injected into the right flank of BALB/c nude mice. Treatments were administered as indicated by the colored triangles (i.p., intraperitoneal). **B** The inhibitory effect of the treatments on relative tumor volume, and images of tumors from NCI-H460/PTX xenograft mice. **C** The tumor inhibition rate (%) and tumor weight (mg) in the different treatment groups. **D** ALDH2 proteins expression levels in NCI-H460/PTX xenograft mice. **E** Representative immunohistochemistry of ALDH2 in NCI-H460/PTX xenograft and statistical analysis of ALDH2-positive cells. **F** Images of tumor sections after the TUNEL assay. The photographs were taken at magnifications of × 200. Scale bar = 50 μm. **G** The inhibitory effect of the different treatment on relative tumor volume, and images of tumors from the NCI-H460/PTX xenograft mice. ^**^p < 0.01, ^***^p < 0.001, compared with control group. ^#^*p* < 0.05, compared with the PTX group. ^&^*p* < 0.05, ^&&^*p* < 0.01, compared with the PTX + DZN group

Our experiments provided information about the molecular mechanisms by which EHMT2 and NFYA cooperatively regulate the expression of ALDH2, and how the EHMT2/NFYA/ALDH2 signaling axis affect the efficacy of PTX in NSCLC/PTX cells. Next, we explored whether downregulating ALDH2 through epigenetic enzyme inhibitors reversed PTX resistance in NSCLC/PTX cells in vitro. Results showed that the combination of PTX and JIB04 at non-cytotoxic concentrations increased the sensitivity of NSCLC/PTX cells to PTX (Fig. S8A-C). Next, we constructed an NCI-H460/PTX xenograft tumor model to investigate whether the combination of JIB04 and PTX can reverse PTX resistance (Fig. 7A). In this model, the JIB04 + PTX group significantly inhibited relative tumor volume and tumor weight compared with the PTX alone group (Fig. 7B and C). Moreover, there was no significant difference in the body weight and viscera index in any of the groups (Fig. S8D-E). The above results suggest that the combination of JIB04 and PTX increased the anti-tumor effect of PTX and reversed the resistance to PTX. The down-regulation of ALDH2 by JIB04 was further confirmed by western blot and immunochemistry in NCI-H460/PTX tumor tissues (Fig. 7D and E). TUNEL staining also confirmed that JIB04 + PTX significantly increased the level of apoptosis in tumor cells (Fig. 7F). Taken together, the above results suggest that the combination of JIB04 and PTX induces cell apoptosis in a PTX-resistant NSCLC xenograft model.

Next, we used the NCI-H460/PTX xenograft tumor model to determine the anti-tumor effect of PTX when an epigenetic approach was used to rescue the expression of ALDH2 (Fig. S8F). One group received PTX + DZN throughout the experiment, one group received PTX + DZN then PTX alone, and one group received PTX + DZN then PTX and the EHMT2 inhibitor UNC0642. Western blot data indicated that the expression of ALDH2 was increased in NCI-H460/PTX tumor tissues when DZN was replaced by UNC0642 (Fig. S8G). As we expected, tumor volume was increased significantly when DZN was withdrawn, because ALDH2 expression recovered gradually to make the tumor cells more resistant to PTX. When DZN was replaced by the EHMT2 inhibitor UNC0642 to upregulate the expression of ALDH2, the tumor volume became bigger than in the other two groups (Fig. 7G). The above results indicated that the expression of ALDH2 can be regulated by an epigenetic approach, which affects the sensitivity of NSCLCs to PTX.

## Discussion

PTX, a disruptor of microtubule dynamics, is used in the treatment of ovarian cancer, breast cancer, lung cancer, head and neck cancer, bladder cancer, esophageal cancer and other malignant tumors [36]. Most patients develop resistance with prolongation of the administration time [37]. Therefore, there is an urgent requirement to explore the molecular mechanism of PTX resistance in order to discover new targets to solve this problem.

In this study, gene microarrays indicated that stemness and drug resistance-related pathways were enriched in NCI-H460/PTX cells compared with NCI-H460 cells. ALDH is widely used as a marker of CSCs [26]. Using the Aldefluor assay, we found that the ALDH activity of NSCLC/PTX cells was significantly higher than that of NSCLC cells. The human ALDH superfamily consists of 19 functional genes with a wide range of tissue expression and substrate specificity [38]. Among ALDH subtypes, the expression of ALDH2 was specifically upregulated. We hypothesized that the upregulation of ALDH2 was significantly correlated with PTX resistance in lung cancer. Our hypothesis was substantiated by data indicating that when ALDH2 expression was modulated by genetic intervention or drug treatment, the responsiveness of NSCLC to PTX changed. In vivo xenograft data indicated that overexpression of ALDH2 in NSCLC cells resulted in a significant increase in tumor growth. Our findings suggest that ALDH2 plays a role as a pro-oncogene in the process of PTX resistance in lung cancer. Similar to our observations, overexpression of ALDH2 resulted in higher cell proliferation rate, higher clone formation rate, and resistance to 4-hydroperoxycyclophosphamide and doxorubicin in leukemia and lung cancer cell lines [24]. Taken together, ALDH2 plays an important role in the process of tumor resistance.

According to our group and other research groups, there are several studies showing that epigenetic enzymes and transcription factors can co-regulate target gene to influence drug resistance. For example, Zhang et al.reported that the histone lysine demethylase KDM6A was recruited to the NTRK1 promoter by the transcription factor YY1, then upregulation of the NTRK1-encoded protein TRKA activated downstream pathways to cause imatinib resistance. The authors identified the KDM6A/YY1/TRKA axis as a new imatinib resistance mechanism in CML [39]. Moreover, our previous studies reported that histone deacetylases (HDAC) and transcription factor retinoic X receptors (RXRs) cooperatively regulated HtrA1 (high temperature requirement factor serine peptidase 1) in cisplatin resistance, and targeting the HDAC/RXR/HtrA1 signaling pathway overcame the cisplatin resistance in NSCLC cells [19]. In present our manuscript, we uncovered that ALDH2 is cooperatively regulated by the histone methyltransferase EHMT2 and the transcription factor NFYA. Taken together, a novel epigenetic/transcription factor/target gene signaling pathway is identified and proven to be involved in drug resistance.

Previous research believed ALDH2 is not only an enzyme involved in aldehyde detoxification but also plays a key role in the growth of tumors. It is reported that ALDH2 influences the removal of endogenous aldehydes generated by the ROS-mediated peroxidation such as 4-hydroxy-2-nonenal (4-HNE), and malondialdehyde (MDA), which are associated with high morbidity of cancer [40, 41]. In addition, ALDH2 promotes the expression of cancer stem biomarkers (e.g., Nanog, Oct4, and Sox2), leading to the proliferation, migration, and invasion of liver cancer stem cells (LCSCs) [42]. However, there are fewer studies investigating how ALDH2 can directly influence the development and progression of tumor. We found that ALDH2 regulated the RAF/MAPK signaling pathway which is involved in many malignant phenotypes, such as cell proliferation, apoptosis, and drug sensitivity [43].Our study demonstrated the efficacy of PTX was decreased when KRAS and RAF1 were overexpressed in ALDH2 knockdown NSCLC/PTX cells, and the effect was similar to that observed when ALDH2 expression was rescued, which established the relationships between the RAS/RAF pathway and ALDH2 in tumors.

At present, corresponding strategies to overcome PTX resistance are as follows: 1) Downregulation of certain transmembrane efflux transporters regulator. For example, knocking down or silencing of FOXO3a decreased P-gp expression [44]; 2) Prevention of microtubule polymerization or microtubule disruption. For example, tubulin inhibitors DJ101 targets the colchicine binding site and prevents the polymerization of tubulin dimers to counteract PTX resistance in PTX-resistant tumors [45]; 3) Regulation of survival/apoptosis pathways related proteins. Betulinic acid has be demonstrated potent anti-cancer activity against PTX resistant H460 lung cancer cells by regulating BCL-2/BAX [46]. However, therapeutic approaches based on these mechanisms have not achieved the promised efficacy. It is particularly important to discover new resistance mechanisms of PTX and corresponding treatment strategies. According to our new mechanism, we propose two strategies to reverse ALDH2-mediated PTX resistance. On the one hand, we use pharmacological inhibitors to directly regulate ALDH2, including a specific inhibitor DZN [47, 48] and non-specific inhibitor DSF [26], an alcohol aversion therapy since the 1940s [49]. We found that DZN + PTX or DSF + PTX had a synergistic effect on the inhibition of cell growth in NSCLC/PTX cells and reduction of NCI-H460/PTX xenograft tumor growth. This indicates that the targeting of ALDH2 reverses PTX resistance. On the other hand, we provided a new way to modulate ALDH2 expression through epigenetic regulators. As we reported before, targeting epigenetic enzymes with specific inhibitors to rescue a tumor suppressor gene or inhibit an oncogene has become a novel therapeutic strategy [50]. In this manuscript, we found that the combination of PTX and JIB04 increased the sensitivity of NSCLC/PTX cells to PTX, and significantly inhibited tumor growth in NCI-H460/PTX xenograft mice compared with PTX alone. However, the synergistic in vivo anti-tumor effect of PTX and DZN/DSF/JIB04 is relatively modest, which might be caused by pharmacokinetics affection and dosage choose. Further study was carrying on in our lab to optimize the dosage and administration methods. Taken together, we believe that combining epigenetic inhibitors and traditional chemotherapy drugs is a bright prospect for overcoming drug resistance and could be used widely in clinical treatment.

## Conclusions

**Fig. 8:**
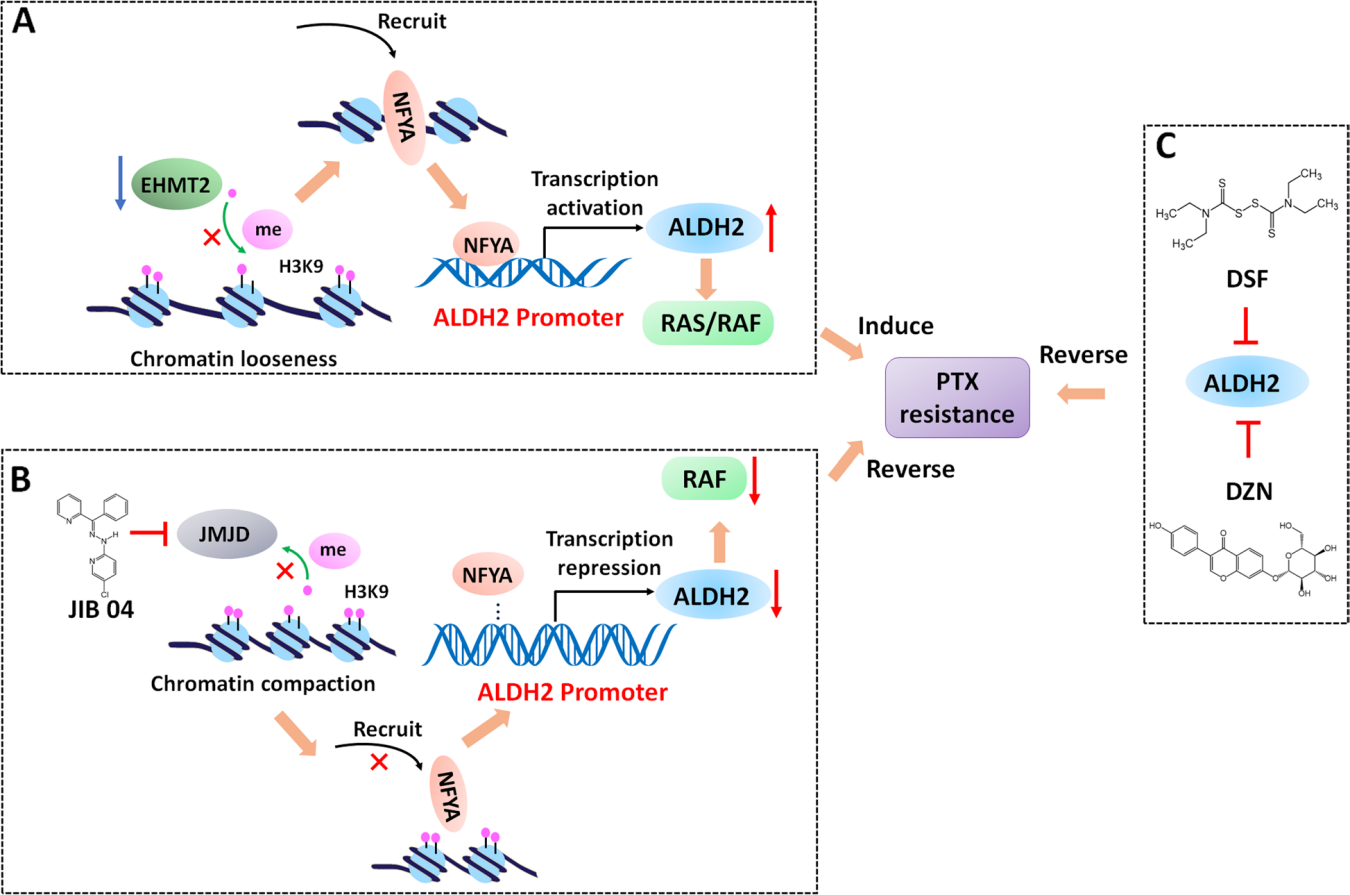
Schematic diagram showing the mechanism of ALDH2-mediated PTX resistance and potential treatment strategies. **A** Regulation of ALDH2 by EHMT2 and NFYA significantly decreases the efficacy of paclitaxel in NSCLC/PTX resistant cells. **B** Epigenetic downregulation of ALDH2 by JIB04 reverses paclitaxel resistance. **C** Pharmacological inhibition of ALDH2 by DZN or DSF reverses paclitaxel resistance

The findings of our study are summarized in a schematic diagram (Fig. 8). The inhibition of EHMT2 and simultaneous overexpression of NFYA upregulate ALDH2 expression, and this signaling axis is involved in mediating resistance to PTX in vitro and in vivo. Mechanistically, NFYA is an important transcriptional activator of ALDH2, and EHMT2 increases the level of H3K9me2 to compact chromatin [51, 52]. Inhibition of EHMT2 promotes binding of NFYA to the ALDH2 promoter, thus activating the transcription of ALDH2. Inhibiting or downregulating ALDH2 by DZN, DSF or JIB04 increases the efficacy of PTX in NSCLC/PTX cells or xenograft tumors and reverses PTX resistance. The results of this study reveal a new strategy to inhibit or downregulate a tumor-promoting gene, which may provide a breakthrough in overcoming PTX resistance. Our work also confirms the role of the epigenetic–transcriptomic in cancers.

## Supplementary Information

**Additional file 1.**

## Data Availability

All data analyzed during this study are included in this published article and its supplementary information files.

## Abbreviations

NSCLC: Non-small-cell lung carcinoma
PTX: Paclitaxel
NFYA: Nuclear transcription factor Y subunit alpha
EHMT2: Euchromatic histone lysine methyltransferase 2
