# Supplementary material for: An EHMT2/NFYA-ALDH2 signaling axis modulates the RAF pathway to regulate paclitaxel resistance in lung cancer
Source: Mol Cancer. 2022 Apr 27;21:106. doi: 10.1186/s12943-022-01579-9 (PMC9044593; doi:10.1186/s12943-022-01579-9)
Supplement: Supplementary file 1 — Additional file 1. [file 12943_2022_1579_MOESM1_ESM.docx]

Supplementary Materials for

**An EHMT2/NFYA-ALDH2 signaling axis modulates the RAF pathway to regulate paclitaxel resistance in lung cancer**

Wenjing Wang ^1,2^, Jianmin Wang ^1,2^, Shuai Liu ^1,2^, Yong Ren ^3^, Jingyu Wang ^1,2^, Sen Liu ^1,2^, Wei Cui ^1^, Lina Jia ^1,2^, Xing Tang ^4^, Jingyu Yang ^1,2^, Chunfu Wu ^1,2,^*, Lihui Wang ^1,2,^*

^1^ Department of Pharmacology, Shenyang Pharmaceutical University, Shenyang, PR China.

^2^ Benxi Institute of Pharmaceutical Research, Shenyang Pharmaceutical University, Shenyang, PR China.

^3^ Department of Pathology, General Hospital of Central Theater Command of People's Liberation Army, Wuhan, PR China.

^4^ Department of Pharmaceutics, Shenyang Pharmaceutical University, Shenyang, PR China.

* Corresponding author.

E-mail addresses: [lhwang@syphu.edu.cn](mailto:lhwang@syphu.edu.cn) (LH Wang), [wucf@syphu.edu.cn](mailto:wucf@syphu.edu.cn%20) (CF Wu)

**The PDF file includes:**

Supplementary Figure 1-8

Supplementary Table 1-2

**SUPPLEMENTAL INFORMATION**


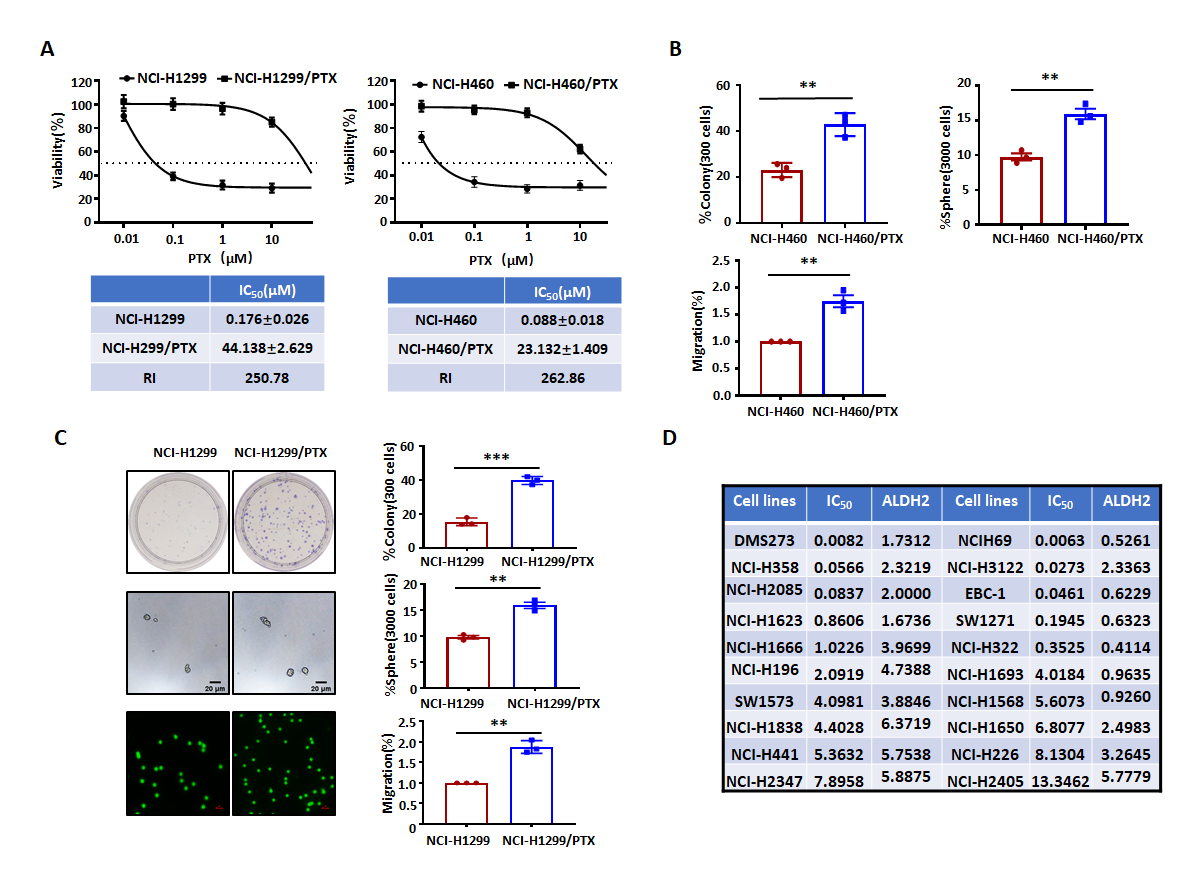


**Supplementary Figure 1. Identification of ALDH2 as a paclitaxel resistance-related gene in NSCLC. (A)** MTT assay results showing the efficacy of PTX in NSCLC and NSCLC/PTX cells. Cells were treated for 72 h. **(B)** Colony formation assay, tumor sphere formation assay and Transwell migration assay in NCI-H460 and NCI-H460/PTX cells. ^**^*p* < 0.01, as compared to parental cells. **(C)** Colony formation assay, tumor sphere formation assay and Transwell migration assay in NCI-H1299 and NCI-H1299/PTX cells. The photographs were taken at magnifications of ×200. Scale bar=50 μm. ^**^*p* < 0.01, ^***^*p*< 0.001, as compared to parental cells. **(D)** The relationship between ALDH2 expression level and PTX resistance in lung cancer cells.


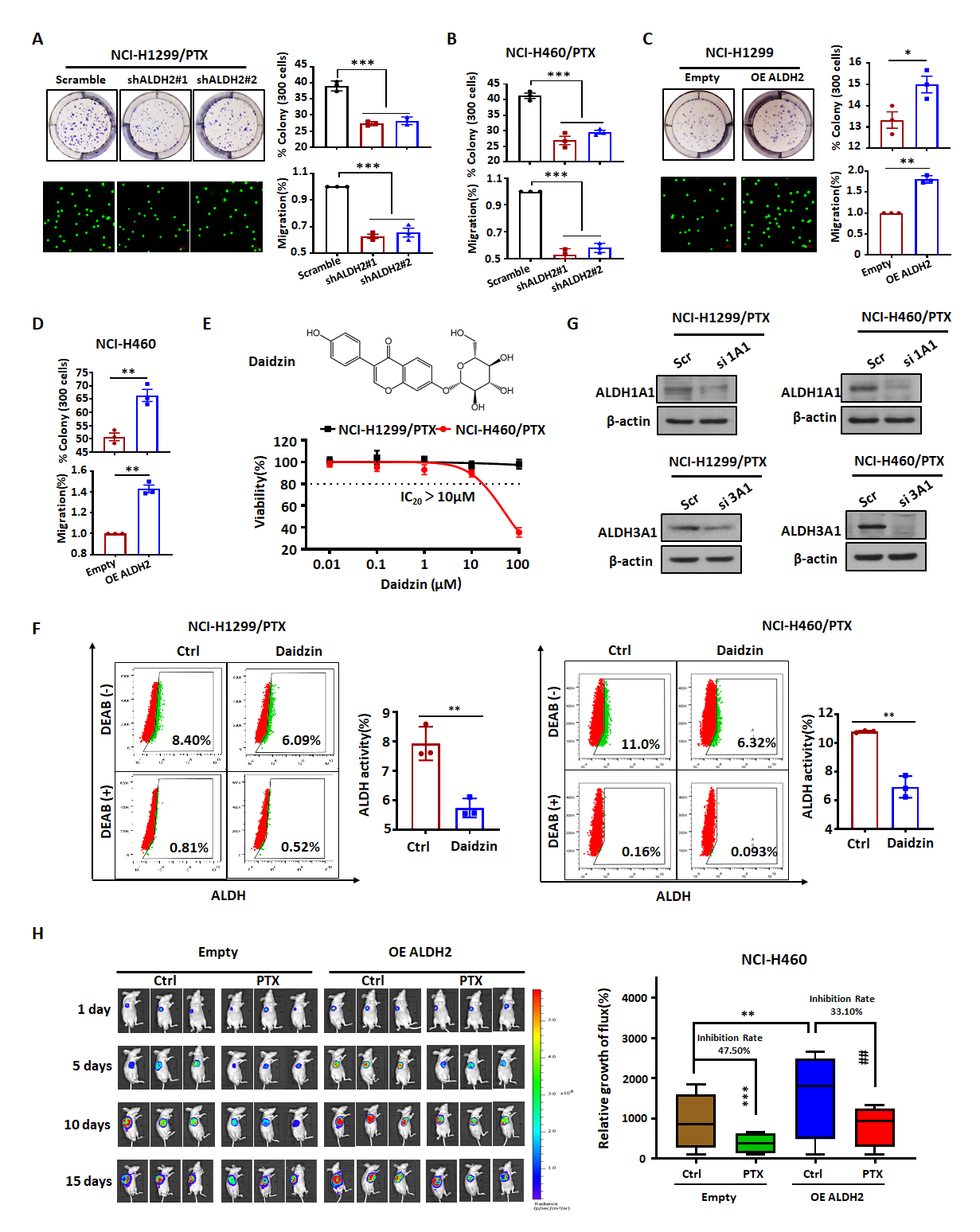


**Supplementary Figure 2. The change in malignant biological characteristics of tumor cells when ALDH2 is regulated by genetic intervention. (A)** Colony formation assay and Transwell migration assay in NCI-H1299/PTX cells transfected with ALDH2 shRNA or scramble. The photographs were taken at magnifications of ×200. Scale bar=50 μm. ^***^*p* < 0.001, as compared to scramble. **(B)** Colony formation assay and Transwell migration assay in NCI-H460/PTX cells transfected with ALDH2 shRNA or scramble. ^***^*p* < 0.001, as compared to scramble. **(C)** Colony formation assay and Transwell migration assay in NCI-H1299 cells transfected with ALDH2 overexpression (OE) or empty plasmid. The photographs were taken at magnifications of ×200. Scale bar=50 μm.^*^*p* < 0.05, ^**^*p* < 0.01, as compared to the empty group. **(D)** Colony formation assay and Transwell migration assay in NCI-H460 cells transfected with ALDH2 overexpression (OE) or empty plasmid. ^*^*p* < 0.05, ^**^*p* < 0.01, as compared to the empty group. **(E)** MTT assay results showing the efficacy of DZN in NSCLC/PTX cells. **(F)** ALDH activity was determined in NSCLC/PTX treated with DZN or vehicle. Cells were labeled with Aldefluor with or without the ALDH inhibitor DEAB and analyzed by flow cytometry. ^**^*p* < 0.01, as compared to the control group. (**G)** The protein expression levels of ALDH subtypes in NSCLC/PTX cells transfected with ALDH siRNA or scramble. **(H)** Bioluminescence images showing tumor growth in NCI-H460 xenograft models treated with PTX or vehicle. ^**^*p* < 0.01, ^***^*p* < 0.001, as compared to the empty group; ^##^*p* < 0.01, as compared to the ALDH2 overexpression group.


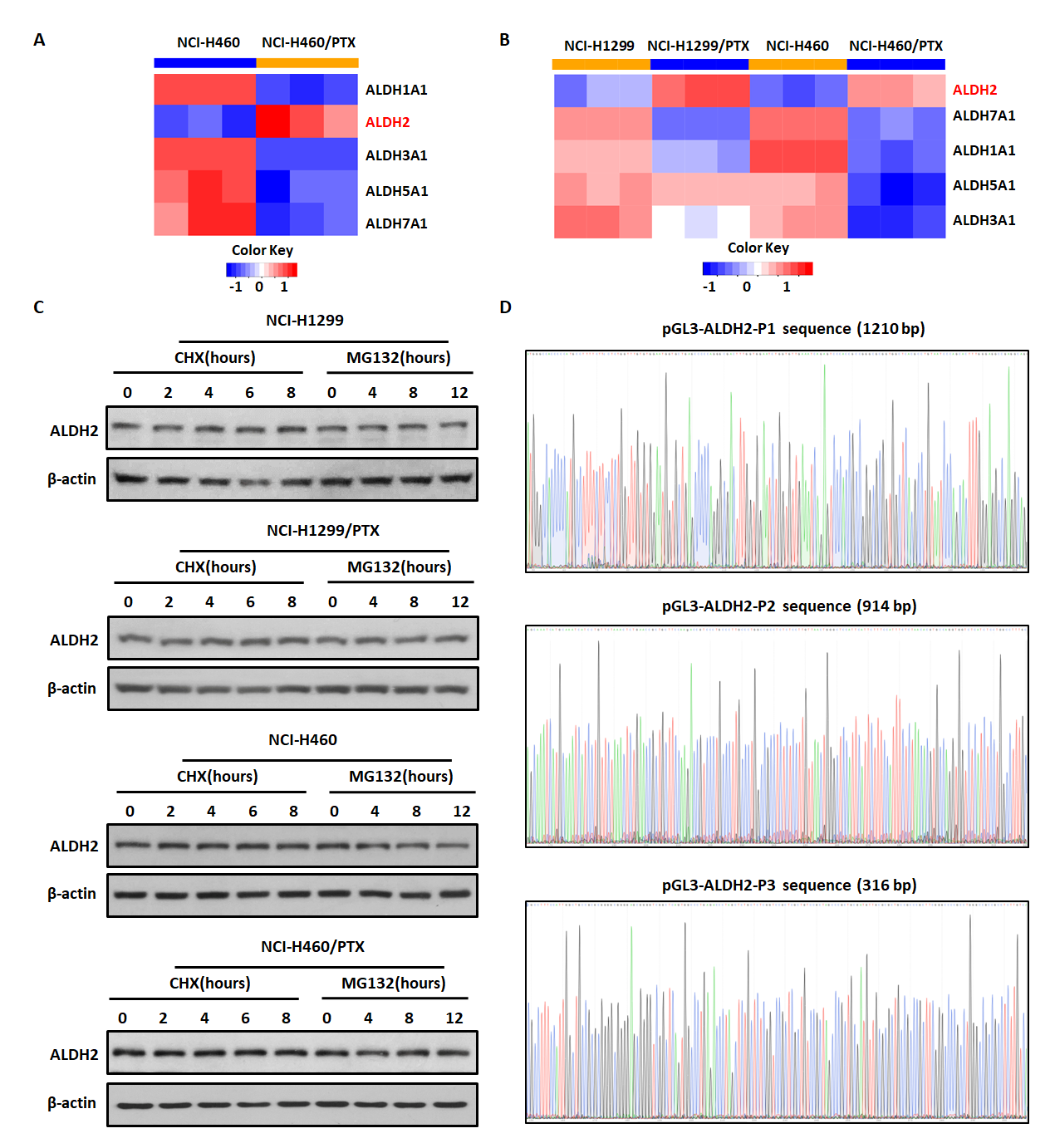


**Supplementary Figure 3. The transcription factor NFYA is involved in the transcriptional activation of ALDH2. (A)** The expression levels of ALDH subtypes in NCI-H460 and NCI-H460/PTX cells as determined by gene microarray analysis. **(B)** Heatmap summarizing the mRNA expression of ALDH subtypes in NSCLC cells and in NSCLC/PTX cells by RT-PCR. **(C)** ALDH2 protein levels in NSCLC cells and NSCLC/PTX cells treated with CHX and MG132. **(D)** Sequencing traces of the PGL3-ALDH2 promoter fragments P1/P2/P3.


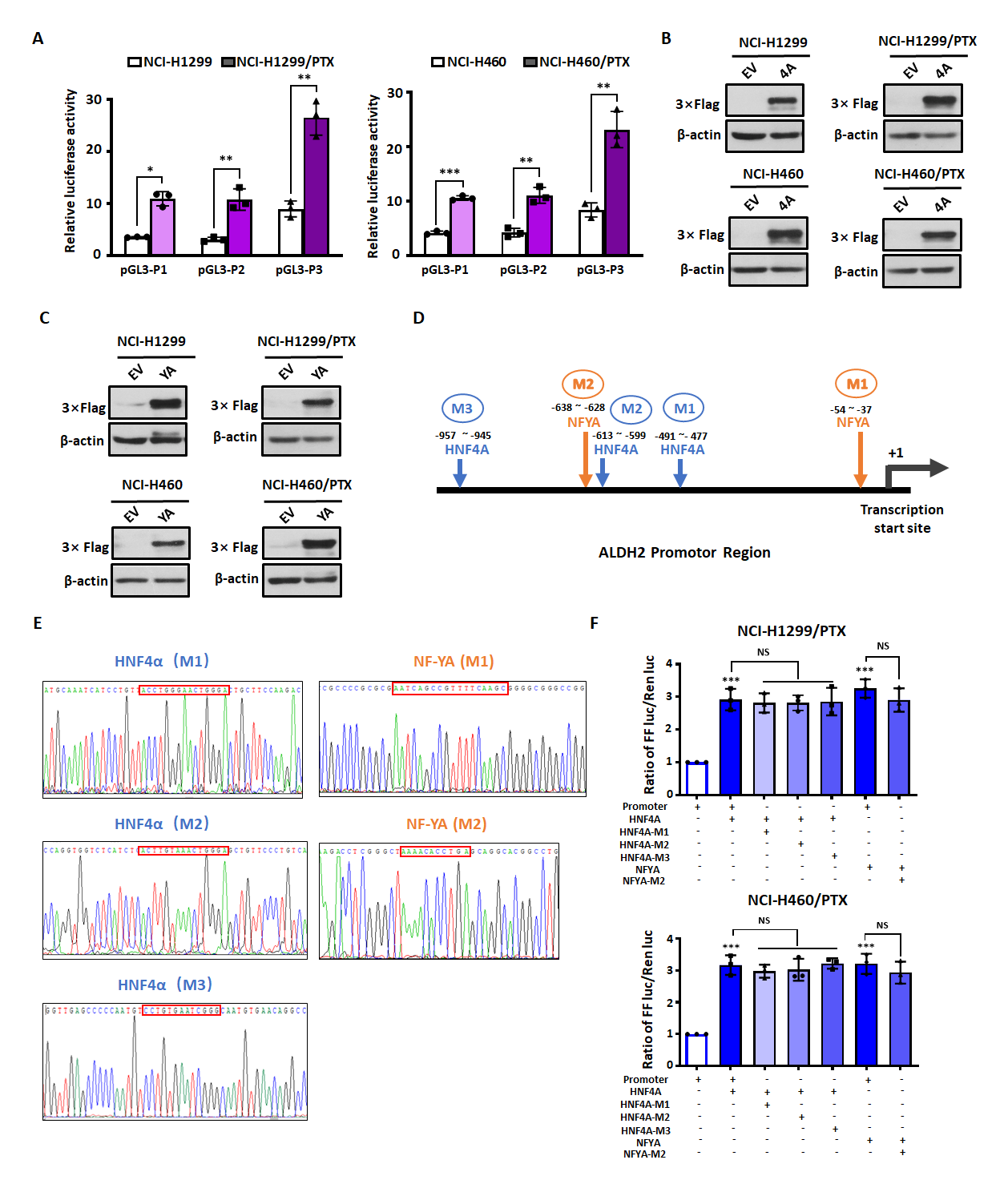


**Supplementary Figure 4. The transcription factor NFYA is involved in the transcriptional activation of ALDH2. (A)** Dual luciferase reporter assay to assess the transcriptional activity of three ALDH2 promoter fragments (P1-P3) in NSCLC cells and NSCLC/PTX cells. ^*^*p* < 0.05, ^**^*p* < 0.01, ^***^*p* < 0.001, as compared to the parental cells group. **(B)** The protein expression levels of HNF4A in NSCLC cells and NSCLC/PTX cells transfected with HNF4A overexpression (4A) or empty (EV) plasmid. **(C)** The protein expression levels of NFYA in NSCLC cells and NSCLC/PTX cells transfected with NFYA overexpression (NY) or empty (EV) plasmid. **(D)** Schematic diagram of the ALDH2 promotor region (P1) showing the location of mutated binding sites for the transcription factors, HNF4A and NFYA. **(E)** Sequencing traces of the mutated HNF4A and NFYA binding sites in the ALDH2 P1 promoter. The resulting constructs are pGL3-HNF4A-M1/M2/M3 and pGL3-NFYA-M1/M2. **(F)** Luciferase activity elicited by the ALDH2 P1 promotor or the indicated binding site mutant (HNF4A-M1/M2/M3 and NFYA-M2) in NSCLC/PTX cells after overexpression of NFYA and HNF4A. ^***^*p* < 0.001, as compared to the promotor group.


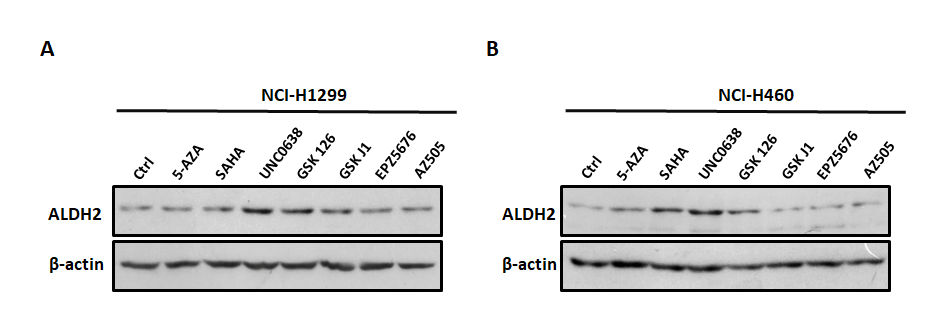


**Supplementary Figure 5. The effect of epigenetic enzyme inhibitor on the transcriptional activation of ALDH2. (A-B)** The protein expression levels of ALDH2 in NSCLC cells treated with epigenetic enzyme inhibitors.


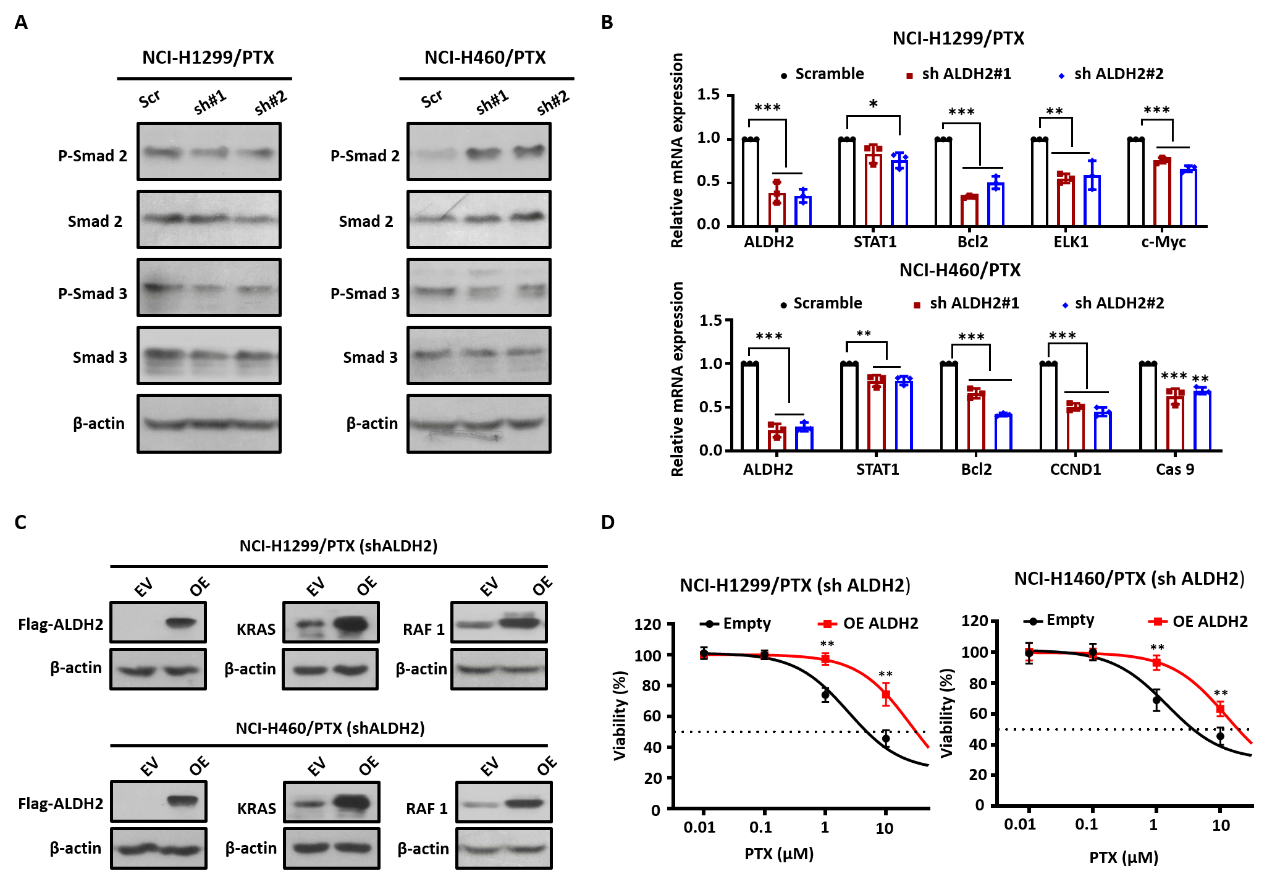


**Supplementary Figure 6. Knockdown of ALDH2 inhibited the RAS/RAF pathway in NSCLC/PTX cells. (A)** The expression levels of proteins related to the TGFβ pathway in NSCLC/PTX cells transfected with ALDH2 shRNA or scramble. **(B)** The mRNA expression levels of genes related to the RAS/RAF pathway in NSCLC/PTX cells transfected with ALDH2 shRNA or scramble. **(C)** The protein expression levels of KRAS, ALDH2 and RAF1 in ALDH2 knockdown NSCLC/PTX cells transfected with KRAS, ALDH2 and RAF1 overexpression (OE) or empty (EV) plasmid. **(D)** MTT assay results showing the efficacy of PTX in ALDH2 knockdown NSCLC/PTX cells transfected with ALDH2 overexpression (OE) or empty plasmid for 72 h. ^*^*p* < 0.05, ^**^*p* < 0.01, ^***^*p* < 0.001 as compared to the empty group or scramble group.


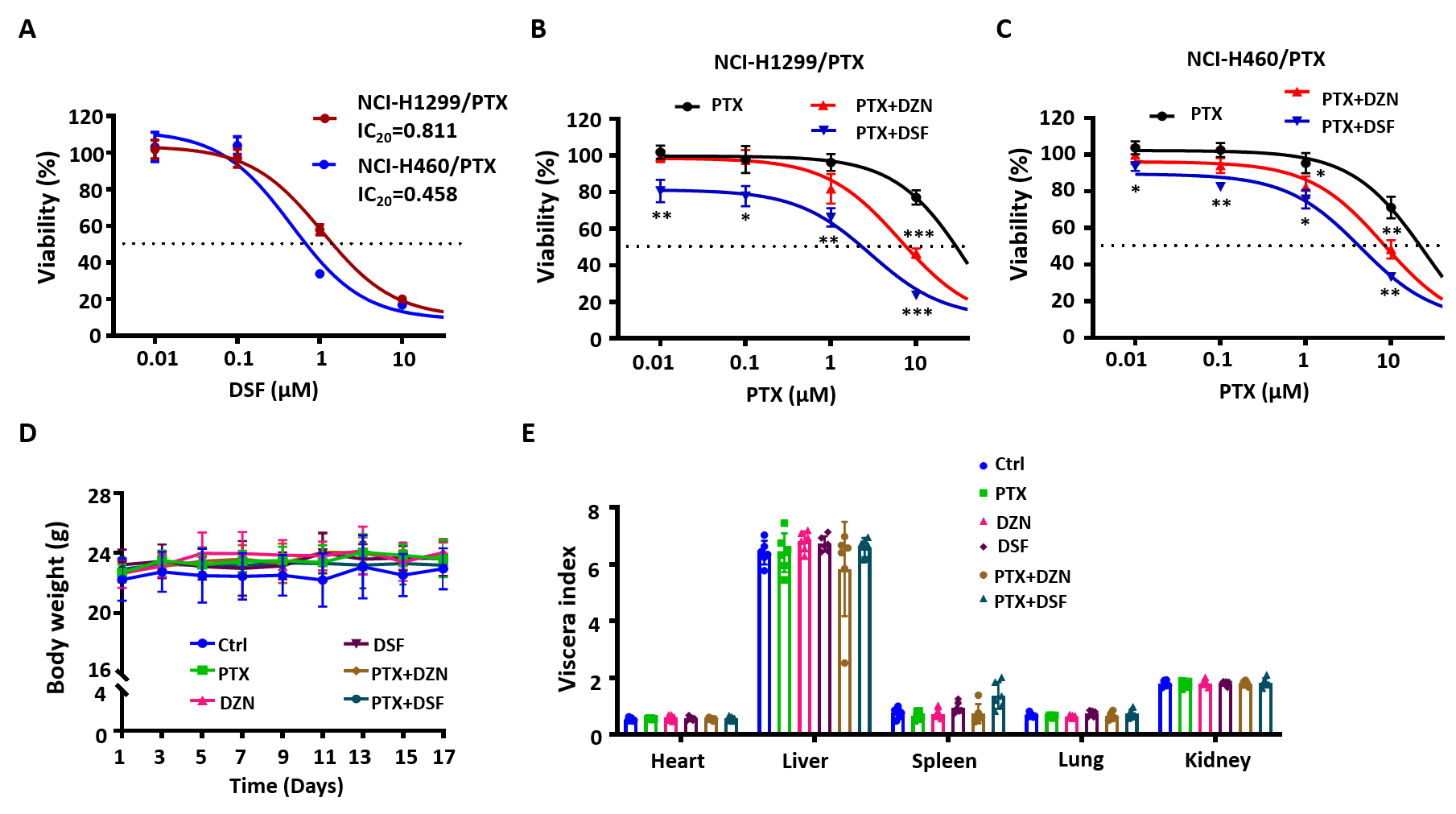


**Supplementary Figure 7. DZN/DSF significantly decreases paclitaxel resistance in NSCLC/PTX cells *in vitro* and *in vivo*. (A)** MTT assay results showing the efficacy of DSF in NSCLC/PTX cells. **(B)** MTT assay results showing the efficacy of PTX in NCI-H1299/PTX cells treated with DZN (10 μM) or DSF (0.8 μM) or vehicle for 72h. **(C)** MTT assay results showing the efficacy of PTX in NCI-H460/PTX cells treated with DZN (10 μM) or DSF (0.4 μM) or vehicle for 72h. ^*^*p* < 0.05, ^**^*p* < 0.01, ^***^*p* < 0.001, compared with PTX group. **(D)** The variation of body weight (g) in the mice from Fig 6a. **(E)** The viscera index in the mice from Fig.6A. ^**^*p* < 0.01, ^***^*p* < 0.001, compared with control group. ^###^*p* < 0.001, compared with the PTX group.

**
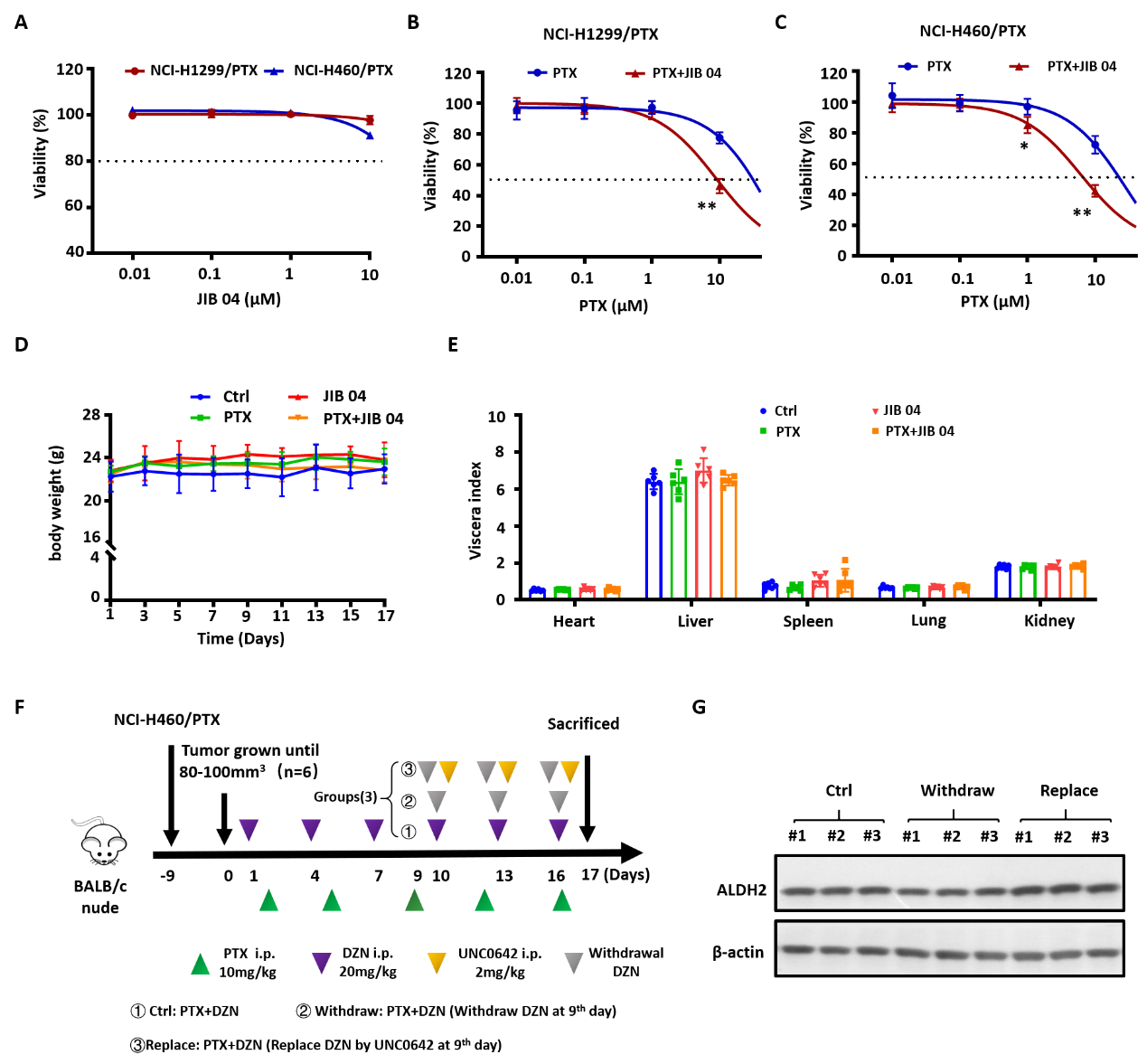
**

**Supplementary Figure 8. JIB04 significantly decreases paclitaxel resistance in NSCLC/PTX cells *in vitro* and *in vivo*. (A)** MTT assay results showing the efficacy of JIB04 in NSCLC/PTX cells. **(B)** MTT assay results showing the efficacy of PTX in NCI-H1299/PTX cells treated with JIB04 (10 μM) or vehicle for 72h. **(C)** MTT assay results showing the efficacy of PTX in NCI-H460/PTX cells treated with JIB04 (10 μM) or vehicle for 72 h. **(D)** The variation of body weight (g) in the mice from Fig. 7a. **(E)** The viscera index in the mice from Fig. 7a. **(F)** Timeline for establishing and treating in NCI-H460/PTX xenograft mice. NCI-H460/PTX cells were subcutaneously injected into the right flank of BALB/c nude mice. Treatments were administered as indicated by the colored triangles (i.p., intraperitoneal). **(G)** The ALDH2 protein levels in NCI-H460/PTX xenograft mice. ^*^*p* < 0.05, ^**^*p* < 0.01, compared with PTX group.

**Supplementary Table 1. Antibody information**

| Antibody | Catalog | Company |
| --- | --- | --- |
| Sox2 | ab92494 | Abcam |
| Nanog | ab109250 | Abcam |
| Oct4 | ab181557 | Abcam |
| ALDH1A1 | NB110-55451 | Novus Biologicals |
| ALDH2 | ab108306 | Abcam |
| ALDH3A1 | NBP-02483 | Novus Biologicals |
| ALDH5A1 | NBP1-32970 | Novus Biologicals |
| ALDH7A1 | NB2-15343 | Novus Biologicals |
| 3×Flag | F3165 | Sigma-Aldrich |
| NFYA | ab139402 | Abcam |
| HNF4A | ab201460 | Abcam |
| H3K9me2 | ab1220 | Abcam |
| Ac-H3-ChIP Grade | ab47915 | Abcam |
| pan RAS | 60309-1-Ig | Proteintech |
| RAF1 | 66592-1- Ig | Proteintech |
| P-MEK1/2 | #9154 | Cell Signaling Technology |
| MEK1/2 | 11049-1-AP | Proteintech |
| P-Erk1/2 | #4370 | Cell Signaling Technology |
| Erk1/2 | #4695 | Cell Signaling Technology |
| NEDD4L | 13690-1-AP | Proteintech |
| Ubiquitin | #3936 | Cell Signaling Technology |
| P-Smad2 | #18338 | Cell Signaling Technology |
| Smad2 | #5339 | Cell Signaling Technology |
| P-Smad3 | sc-517575 | Santa Cruz |
| Smad3 | sc-101154 | Santa Cruz |
| β-actin | sc-8432 | Santa Cruz |

**Supplementary Table 2. Primer Sequences**

| Gene | Forward | Reverse |
| --- | --- | --- |
| ALDH2 | TGTGTGGGTCAACTGCTATGA | TCACTTCAGTGTATGCCTGCA |
| ALDH1A1 | GCTGGCGACAATGGAGTCAATG | CGTGGAGAGCAGTGAGAGGAGT |
| ALDH3A1 | GATGCCGCCACTCGCTACATAG | GGCTTCTCACGCTGGTTGATGA |
| ALDH5A1 | CCGCCAAGGAGAGGAGTTCATT | GCAGGCTTCACCACGACAGT |
| ALDH7A1 | GGAACAACGCCATCGCCATGA | AACAGTCGCCTCGCAGTGGTA |
| KRAS | GTAGTTGGAGCTGGTGGCGTAG | ACTCCTCTTGACCTGCTGTGTC |
| RAF1 | CACAGCGAATCAGCCTCACCTT | GCAGAACAGCCACCTCATTCCT |
| STAT1 | CTTCCTGCTGCGGTTCAGTGAG | TCCATTGGCTCTGGTGCTTCCT |
| Bcl2 | TTCGCCGAGATGTCCAGCCA | GCATCCCAGCCTCCGTTATCCT |
| ELK1 | CCACCTTCACCATCCAGTCTCT | CCGCAACTTCCAACTCTTCCTT |
| c-Myc | CGTCCTCGGATTCTCTGCTCTC | GCTGCGTAGTTGTGCTGATGTG |
| CCND1 | TACCGCCTCACACGCTTCCT | TCCACCTCCTCCTCCTCCTCTT |
| Cas9 | CGAACTAACAGGCAAGCAGCAA | TCAAGAGCACCGACATCACCAA |
| β-actin | TCGTGCGTGACATTAAGG | AAGGAAGGCTGGAAGAGT |
| ALDH2-ChIP | ACCCGCTTCGCTTGCATCAG | AGAGCAGAGCTAGGGTCTCAGG |
